# Skeletal Muscle-Restricted Expression of Human SOD1 in Transgenic Mice Causes a Fatal ALS-Like Syndrome

**DOI:** 10.3389/fneur.2020.592851

**Published:** 2020-12-14

**Authors:** Lee J. Martin, Margaret Wong

**Affiliations:** ^1^Division of Neuropathology, Department of Pathology, Johns Hopkins University School of Medicine, Baltimore, MD, United States; ^2^Pathobiology Graduate Training Program, Johns Hopkins University School of Medicine, Baltimore, MD, United States; ^3^Department of Neuroscience, Johns Hopkins University School of Medicine, Baltimore, MD, United States; ^4^Department of Anesthesiology and Critical Care Medicine, Johns Hopkins University School of Medicine, Baltimore, MD, United States

**Keywords:** rapsyn, motor neuron, DNA damage, aging, TDP-43

## Abstract

Amyotrophic lateral sclerosis (ALS) is a fatal heterogeneous neurodegenerative disease that causes motor neuron (MN) loss and skeletal muscle paralysis. It is uncertain whether this degeneration of MNs is triggered intrinsically and is autonomous, or if the disease initiating mechanisms are extrinsic to MNs. We hypothesized that skeletal muscle is a primary site of pathogenesis in ALS that triggers MN degeneration. Some inherited forms of ALS are caused by mutations in the *superoxide dismutase-1* (*SOD1*) gene, that encodes an antioxidant protein, so we created transgenic (tg) mice expressing wild-type-, G37R-, and G93A-human *SOD1* gene variants only in skeletal muscle. Presence of human SOD1 (hSOD1) protein in skeletal muscle was verified by western blotting, enzyme activity gels, and immunofluorescence in myofibers and satellite cells. These tg mice developed limb weakness and paresis with motor deficits, limb and chest muscle wasting, diaphragm atrophy, and age-related fatal disease with a lifespan shortening of 10–16%. Brown and white adipose tissue also became wasted. Myofibers of tg mice developed crystalline-like inclusions, individualized sarcomere destruction, mitochondriopathy with vesiculation, DNA damage, and activated p53. Satellite cells became apoptotic. The diaphragm developed severe loss of neuromuscular junction presynaptic and postsynaptic integrity, including decreased innervation, loss of synaptophysin, nitration of synaptophysin, and loss of nicotinic acetylcholine receptor and scaffold protein rapsyn. Co-immunoprecipitation identified hSOD1 interaction with rapsyn. Spinal cords of tg mice developed gross atrophy. Spinal MNs formed cytoplasmic and nuclear inclusions, axonopathy, mitochondriopathy, accumulated DNA damage, activated p53 and cleaved caspase-3, and died. Tg mice had a 40–50% loss of MNs. This work shows that hSOD1 in skeletal muscle is a driver of pathogenesis in ALS, that involves myofiber and satellite cell toxicity, and apparent muscle-adipose tissue disease relationships. It also identifies a non-autonomous mechanism for MN degeneration explaining their selective vulnerability as likely a form of target-deprivation retrograde neurodegeneration.

## Introduction

Amyotrophic lateral sclerosis (ALS) is a fatal neurodegenerative disease that causes skeletal muscle paralysis, respiratory failure, and death generally within 3–5 years after symptom onset (1, 2). Muscle weakness and fasciculations are early clinical signs. The cause of the morbidity is progressive skeletal muscle loss of function and degeneration of upper motor neurons (MNs) in cerebral cortex and lower MNs in brainstem and spinal cord (2, 3). Aging and heterogeneous gene mutations are risk factors for ALS. Most ALS cases are sporadic with no known inheritance pattern. The driving molecular mechanisms of the disease are unknown, and there are no effective treatments (2). Inherited or familial forms of ALS (fALS) have autosomal dominant or autosomal recessive patterns and make up ~10% or less of all ALS cases (2, 4). Heterozygous mutations in the *superoxide dismutase-1* (*SOD1*) gene account for ~20% of all fALS cases (~2% of all ALS cases) (5, 6). SOD1 (copper/zinc SOD) is ubiquitous in most tissues (7) and is a metalloenzyme that functions as a ~32 kDa non-covalently assembled homodimer of ~16 kDa subunits that bind one copper ion and one zinc ion (8). This enzyme detoxifies and maintains intracellular superoxide anion (O_2_^−^) concentration in the low femtomolar range by catalyzing the dismutation of O_2_^−^ to molecular oxygen and hydrogen peroxide (8). SOD1 mutants appear to gain a toxic property or function, rather than losing O_2_^−^ scavenging activity (5, 9, 10), and wild-type SOD1 can become toxic through oxidative post-translation modification and zinc deficiency (11–14).

Nearly three decades have elapsed since the discovery of hSOD1 linkage to fALS (5, 6); yet, the triggering events for MN injury and degeneration in ALS are still not understood, and the apparent early preferential vulnerability of MNs remains unexplained. Many etiologies have been implicated, including neurotrophin deprivation, axonopathy, neuronal hyperexcitability and excitotoxicity, DNA damage, nuclearopathy, protein aggregation and proteasome dysfunction, mitochondriopathy, and oxidative stress (2, 15–17). Many of these mechanisms are consistent with SOD1 biology and its proneness to proteinopathy and aberrant oxidative chemistry being disease drivers (18). Notwithstanding, the extent to which specific intrinsic abnormalities within MNs and other spinal cord cells contribute to the pathogenesis of ALS is controversial. MN degeneration was not observed in transgenic (tg) mice expressing human mutant SOD1 selectively in neurons by a Thy1 promoter (19, 20); however, neuron-specific expression of human mutant SOD1 was sufficient to induce MN degeneration in other tg mice (21, 22). Tg mice with astrocyte-specific expression of human mutant SOD1 did not develop disease (23), but cell culture studies indicate that mutant SOD1 in astrocytes can cause degenerative changes in wild-type MNs and worsen toxicity of mutant SOD1 within MNs (24). Human mutant SOD1 expressed in microglia might also be toxic to MNs in tg mice (25, 26) and in cell culture (27); furthermore, degeneration of MNs expressing mutant SOD1 in wild-type astrocyte or microglia environments delayed or prevented in chimeric mice (28) and in mice with wild-type neural progenitor cell transplants (29). However, elimination of proliferating microglia-expressing human mutant SOD1 in mice did not affect MN degeneration (30).

Cells and tissues outside the CNS are diseased in human ALS, including lymphocytes (31), fibroblasts (32, 33), skeletal muscle (34, 35), and adipose (36). Emerging evidence suggests a negative systemic metabolic syndrome in ALS (37, 38), with a third to about half of all ALS patients having hypermetabolic resting energy expenditure (39, 40). Skeletal muscle pathology and early functional abnormalities occur in patients with sporadic ALS (41–43) and fALS (44, 45) and in mutant SOD1 tg mice (38, 46, 47), but abnormalities in skeletal muscle are interpreted often as secondary to CNS disease (42, 48). However, tg mice expressing ALS-causing mutant hSOD1 in only skeletal muscle develop motor deficits and skeletal muscle disease involving myofiber atrophy and mitochondrial perturbations (49) and also oxidative stress and increased protein nitration and MN degeneration, even with low copy number of transgene (50). These latter mice were examined at young to mid-life, but critical later-life phenotype assessments were not done (50) to fully test the hypothesis that skeletal muscle is a primary site of pathogenesis that manifests as an ALS-like disease. This study shows that aged tg mice expressing wild-type-, G37R-, and G93A-human *SOD1* gene variants only in skeletal muscle develop a fatal ALS-like disease phenotype involving prominent skeletal muscle and spinal cord pathology, suggesting a skeletal muscle disease triggered, MN non-autonomous degeneration in ALS driven by DNA damage and retrograde neurodegeneration.

## Materials and Methods

### Transgene Design and Creation of hSOD1^mus^ tg Mice

All mouse experiments complied with regulations of the Animal Care and Use Committee at Johns Hopkins University School of Medicine in accordance with the laws of the State of Maryland and the United States of America. A 247 bp ClaI/XmaI fragment containing the chicken skeletal muscle α_sk_ actin promoter from plasmid CLA12-191αACTCAT1 (51) was cloned into the ClaI/XmaI multiple cloning site of the pBluescript IISK(+) phagemid (Stratagene). This short xeno-promoter has been used to create several lines of tg mice with skeletal muscle-specific expression of exogenous proteins (51–54). The G37R and G93A point mutations were introduced into the wild-type (WT) hSOD1 gene coding sequence within entry clone IOH4089 (Invitrogen) using the QuikChange II Site-Directed Mutagenesis kit (Stratagene). PCR was used to amplify wild-type and mutant hSOD1 cDNA sequences from the entry clones using 5′and 3′ primers flanked by BamHI restriction sites. The 3′ primer also contained ATTAAA, the poly adenylation signal 5′ to the BamHI site. The amplified 555 bp product was cloned into the BamHI cloning site in pBluescript that was 3′ to the chicken α_sk_ actin promoter (51). All sequences were directly confirmed by sequencing in both forward and reverse directions.

To create tg mice, the 3 different transgene-containing plasmids were digested with ApaLI, resulting in a 2.5 Kb fragment. The fragments were given to the Johns Hopkins Transgenic Core Facility for injection into B6SJLF1 mouse embryos. Tg mouse positives were confirmed by PCR analysis of tail genomic DNA using 3 different primer pairs. Mouse tails were digested using DirectPCR Lysis Reagent (Viagen Biotech) and DNA was extracted from the lysate by precipitation with isopropanol. The presence of the transgene was confirmed by PCR using primers described (50). All PCR products contained the coding region of the hSOD1 gene. Southern blot analysis was used to determine the presence of transgene and copy number. In this study, the tg mouse lines used for longitudinal assessments were: G37R-hSOD1^mus^ (lines 25 and 73); G93A-hSOD1^mus^ (lines 98 and 112); and WT-hSOD1^mus^ (lines 125 and 224). The mouse generations were F9, F10, and F11.

### Assessment of Neurologic Deficits in hSOD1^mus^ tg Mice

Tg hSOD1^mus^ mice and age-matched non-tg littermate controls were examined longitudinally beginning at the day of birth to the end of their lives. Older mice were checked daily in the vivarium. All mice were barcoded and genotyped from tail genomic DNA at 1 month of age. They were assessed for neurologic deficits beginning at 1 month of age until life ending. Neurologic test results from 6 to 12 months of age have been reported (50). Here, we report data from 1 to 2 years of age. Mice were assessed using a wire hang-time test, as described (50), and a swimming test. The swimming test is a novel assessment. A plastic rectangular pool (14” × 17”) filled with 3.5” of water (35–40°C) was used. This water level was enough to allow mice to climb out of the pool onto side of the pool. The bottom of the pool was divided into four quadrants. Test mice were placed gently by their tail into the center of the pool (identified by intersecting lines on the pool bottom) and were videotaped by a central overhead camera for 2-min episodes. Swimming videos were evaluated and scored on a point range of +25 (best possible) to −25 (worst possible). Mice were assigned positive scores for using individual limbs and corresponding negative scores for not using individual limbs; similarly, positive scores for time in active swimming or negative scores for body freezing/floating. Coordinated tail use for swimming was also scored. If the mice were observed to be struggling and at risk for submersion, they were quickly removed from the pool. If mice were at risk of submersion immediately after being placed into the pool, they were quickly removed and assigned a score of −25. The tests and analyses were done blinded to genotype. At least 2 trials were done for each mouse and then averaged.

### Anatomical Pathology

All mice had thorough necropsies, including mice that were killed for fresh tissue harvesting and those that were perfusion-fixed with 4% paraformaldehyde and used for anatomical pathology studies. Mice were examined grossly externally and internally for evidence of malignancy. They were stripped of their skin, and chest circumference was measured (in mm) consistently at the manubrium articulation with the xyphoid process. Interscapular white adipose tissue (WAT) and the underlying brown adipose tissue (BAT) located deep between the scapulae were collected and weighed. The hindlegs were cut from the body precisely between the two trochanters, felt by palpation on the femur, and weighed. The diaphragms were carefully cut using iridectomy scissors away from the vertebra, costal margins, and xyphoid process, weighed, and broadest point diameters were measured including left and right muscle flanks and central tendon. The brains and spinal cord were removed from the skull and vertebral column intact from the frontal poles anteriorly to the spinal cord conus posteriorly. The spinal cord was transected at cervical level C1 and weighed.

### Nitric Oxide (NO) Tracking in Skeletal Muscle

NO production in myofibers *in vivo* was tracked using 1,2-diaminoanthraquinone (DAA, Invitrogen Molecular Probes). DAA is a non-fluorescent aromatic vicinal diamine that reacts selectively with NO to yield a fluorescent product (55, 56). Tracer was prepared in Influx (Invitrogen Molecular Probes) pinocytic cell-loading reagent. Myofibers were loaded *in vivo* by injection of DAA (50 μM, 10 μl boluses) into the gastrocnemius along its midline length. Injections (100 μl boluses) were also made into the thoracic cavity through the intercostal muscles. DAA with loading reagent is endocytosed (57, 58). One day later mice were overdosed with anesthesia and perfused with paraformaldehyde. As a negative control, N^G^-nitro-L-arginine methyl ester (L-NAME) was injected (50 mg/kg, ip) to inhibit all forms of nitric oxide synthase (NOS). Gastrocnemius muscles were cryoprotected and frozen sectioned transversely into 40 μm sections that were mounted on glass microscope slides. Diaphragms were evaluated as whole mounts. NO histochemical preparations were analyzed for fluorescence intensity quantitatively using methods described (59, 60).

### Immunoblotting, Immunoprecipitation, Biochemistry, and In-Gel Activity Assays

Western blot analysis was done on skeletal muscles and spinal cord to examine the levels of several target proteins (Table 1), including hSOD1 and other proteins broadly categorized as markers for the neuromuscular junction (NMJ), MNs and their injury response, cell death and DNA damage response (DDR), and oxidative stress. hSOD1^mus^ tg mice 1.5–2.2 years old (*n* = 18 for each genotype) and age-match non-tg littermate control mice (*n* = 20) were used. Some comparisons also included tissues from mice (*n* = 20 per genotype) <1.5 years of age as reported before (50). The mice received a lethal dose of anesthetic and were decapitated for harvesting forelimb and hindlimb skeletal muscle (triceps, quadriceps femoris, biceps femoris, gastrocnemius, and tibialis anterior), diaphragm, brain, and spinal cord which were quickly frozen on dry ice.

**Table 1:** Primary antibodies used.

**Target protein**	**Source**	**Assay**
SOD1	Stressgen	Western blotting (WB)
Human SOD1	MBL International, clone1G2	Immunoprecipitation (IP), immunohistochemistry (IHC)
TDP-43	ProteinTech	IHC
SOD2	Stressgen	IHC
Phospho-p53^Ser15^	R&D systems	WB, IHC
Cleaved caspase-3	R&D systems	IHC
ChAT	Chemicon-Millipore	WB
Neurofilament	Covance, SMI-32	IHC
Synaptophysin	Abcam	WB
nAChR	ThermoFisher, clone 88B	WB
Rapsyn	Lifespan Bioscience	WB, IP
Calcineurin	MBL	WB, IP
MyoD	Developmental studies hybridoma bank	IF
Pax7	Developmental studies hybridoma bank	IF
3-Nitrotyrosine	Abcam	WB, IP
NOS1	BD Transduction laboratories	WB
NOS3	BD Transduction laboratories	WB
Ubiquitin	Sigma	IHC

These samples were minced or pulverized and homogenized with a Brinkmann polytron in ice-cold 20 mM Tris HCl (pH 7.4) containing 10% (wt/vol) sucrose, 200 mM mannitol, complete protease inhibitor cocktail (Roche), 0.1 mM phenylmethylsulfonyl fluoride, 10 mM benzamidine, 1 mM EDTA, and 5 mM EGTA. Crude homogenates were sonicated for 15 s and then centrifuged at 1,000 g_av_ for 10 min (4°C). The supernatant was centrifuged at 54,000 g_av_ for 20 min (4°C) to yield soluble (S2) and membrane-enriched pellet (P2) fractions. This subcellular fractionation protocol has been verified (61). The pellet fraction was washed (twice) by trituration in homogenization buffer followed by centrifugation and then finally resuspended in homogenization buffer (without sucrose) supplemented with 20% (wt/vol) glycerol. Protein concentrations were measured by a Bio-Rad protein assay with bovine serum albumin as a standard.

Proteins from skeletal muscle and spinal cord were subjected to sodium dodecyl sulfate polyacrylamide gel electrophoresis (SDS-PAGE) and transferred to nitrocellulose membrane by electroelution as described (61). For immunoprecipitation (IP) prior to SDS-PAGE, skeletal muscle protein extract (500 μg) was input for 5 μg hSOD1 antibody (Table 1) followed by agarose-conjugated protein A (Pierce) for capture. Negative control conditions for IP experiments were homogenates from non-tg mice, IP with isotype specific non-immune IgG, IP with PBS, and IP with no homogenate input. Final IP samples were subjected to SDS-PAGE and western blot. The reliability of sample loading and electroblotting for every blot was evaluated by staining nitrocellulose membranes with Ponceau S before immunoblotting. If transfer was not uniform, blots were discarded, and gels were run again. Ponceau S stained membranes were imaged and used as protein loading controls. Blots were blocked with 2.5% non-fat dry milk with 0.1% Tween 20 in 50 mM Tris-buffered saline (pH 7.4), then incubated overnight at 4°C with primary antibody (Table 1). The antibodies were used at concentrations for visualizing protein immunoreactivity within the linear range as described (62). After primary antibody incubation, blots were washed, and incubated with horseradish peroxidase-conjugated secondary antibody (0.2 μg/ml), developed with enhanced chemiluminescence (Pierce), and exposed to x-ray film or digital imaging. After careful verification of immunoreactive band identity using positive and negative controls, immunoreactivities of target proteins were measured by densitometry.

The biochemical activity for catalase was determined in skeletal muscle extracts using a spectrophotometric assay (Sigma-Aldrich). Soluble protein fractions from age-matched non-tg mice and hSOD1^mus^ tg mice (*n* = 6/group) at 1.5 years of age were used as input. Bovine catalase was used as a positive control. Copper sulfate was used as a catalase inhibitor negative control. Enzymatic reactions were read at 520 nm.

For in-gel SOD activity assays, native SDS-PAGE was done on skeletal muscle extracts from tg and non tg mice. Purified human SOD1 (Sigma) was loaded as a positive control. A two-step procedure using nitroblue tetrazolium and riboflavin was done (63). Potassium cyanide was used as an inhibitor to determine the specificity of enzyme visualization.

### Histology and Immunohistochemistry

After perfusion-fixation, forelimb triceps muscle, hindlimb biceps femoris muscle, and CNS were removed from each mouse, and the tissues were cryoprotected (20% glycerol) before they were frozen-sectioned (40 μm) using a sliding microtome. Serial tissue sections were stored individually in 96-well plates. The diaphragm was removed intact for studying as a whole mount. Skeletal muscle sections were used for terminal deoxynucleotidyl nick-end labeling (TUNEL) to visualize DNA damage (DNA double-strand breaks)-cell death as described (59, 64) and immunofluorescence to study the localizations of hSOD1 and satellite cell markers (65) as described (60, 66). Spinal cord sections were used for Nissl (cresyl violet, CV) and silver staining, TUNEL, and immunohistochemistry (IHC). CV staining was done on every 10th section of lumbar and cervical spinal cord. Silver staining was used to visualize degenerating neurons in every 11th spinal cord section using the FD NeuroSilver kit (FD Neurotechnologies Inc, Baltimore, MD). Immunoperoxidase IHC, with diaminobenzidine (DAB) as chromogen, was done on spinal cord sections to localize ubiquitin, TDP-43, SOD2, phospho-p53, and cleaved caspase-3. Counterstaining with CV was done for cellular and neuroanatomical perspective.

### Cell Counting and Pathology Measurements

Profile counting was used to estimate the numbers of: [1] TUNEL-positive nuclei in skeletal muscle and spinal cord ventral horn; [2] morphologically normal spinal MNs in CV-stained sections; [3] chromatolytic MNs and axonal swellings in TDP-43 IHC sections; [4] cleaved caspase-3- and phospho-p53-positive MNs in IHC sections; and [5] MN perikaryal mitochondria in SOD2 IHC sections. For all counting, sections were selected with a random start and then systematically sampled (every 10th section) to generate a subsample of sections from each mouse muscle or spinal cord that were mounted on glass slides for evaluation. MNs were counted at 400x magnification using strict morphological criteria. These criteria included a round, open, pale nucleus (not condensed and darkly stained), globular Nissl staining of the cytoplasm, and a diameter of ~25–40 μm. With these criteria, astrocytes, oligodendrocytes, and microglia were excluded from the counts. Axonal swellings were counted in the lumbar spinal cord ventral funiculus at 400x. MN perikaryal SOD2-positive mitochondria were counted at the base of the primary dendrites near the nucleus at 1000x.

### Neuromuscular Junction (NMJ) Analysis

To study the NMJ, motor endplates in diaphragms were visualized using fluorescent-conjugated α-bungarotoxin (BTX) that binds irreversibly to postsynaptic acetylcholine receptors on the skeletal muscle plasma membrane (67). Motor endplates were visualized with Alexa 594-conjugated α-BTX (Invitrogen) as described (29). MN axons were visualized in two ways. We generated double tg mice by crossing hSOD1^mus^ tg mice with B6.Cg-tg Hlxb9-gfp1^Tmj/j^ mice expressing eGFP driven by the mouse Hb9 promoter (68). Dual labeling was also done to visualize MN distal axons and their synaptic terminals in skeletal muscle by immunofluorescent detection of either neurofilament protein or synaptophysin (Table 1). The immunofluorescent labeling for neurofilament was used to determine whether the endplates were innervated. Sections were analyzed using a Zeiss Axiphot epifluorescence microscope or an LSM 410 confocal microscope. The BTX staining patterns were used to assess quantitatively endplate structure in age-matched hSOD^mus^ tg and non-tg mice. Endplates were scored as innervated or denervated.

### Comet Assay

We used the comet assay to measure DNA damage in diaphragm myonuclei and spinal cord MN nuclei of hSOD1^mus^ tg mice and age-matched controls. Whole diaphragm cell nuclei were isolated from fresh tissue as described (66). MN nuclei were isolated from fresh lumbar spinal cord using differential centrifugation as described (69, 70). The alkaline comet assay was used to detect DNA single-strand breaks (DNA-SSBs) and the percentage of nuclei with comet tails as described (69, 70).

### Electron Microscopy (EM)

Age-matched non-tg and hSOD1^mus^-G37R, -G93A, and -wild-type tg mice (15–17 months of age) received an anesthetic overdose and were perfused transcardially with 2% paraformaldehyde/2% glutaraldehyde. The group sizes were 2 mice per genotype. Tissue samples of left and right biceps femoris were taken from each mouse and processed and embedded in plastic for conventional transmission EM as described (71). Tissue samples were cut in the transverse plane at 0.5 μm for high-resolution light microscopy at 1000x magnification, and then thin sections were cut and collected on copper grids for EM. These sections were viewed and imaged using a Phillips CM12 electron microscope. Digital electron micrographs from each mouse genotype were used to examine and quantify myofiber sarcomeres, inclusions, mitochondria, and satellite cells in at least twenty images per mouse.

### Data Analysis

Analyses were done using Excel and GraphPad Prism 8. Group means and variances were evaluated statistically by one-way ANOVA and a Newman-Keuls *post-hoc* test or a Kruskal-Wallis one-way analysis of variance by ranks and a *post-hoc* Dunn's multiple comparison test. *P* values of < 0.05 were considered significant.

### Photography and Figure Construction

The original images used for figure construction were generated using digital microscopic photography or scanning x-ray films. Digital images from the microscope were captured as TiF files using a SPOT digital camera and SPOT Advanced software (Diagnostic Instruments) or a Nikon digital camera (DXM1200) and ACT-1 software. Images were adjusted for brightness and contrast using Adobe Photoshop software without changing the content and actual result. Figure composition was done using CorelDraw Suite 2019 software with final figures being converted to TiF files. Files of composite figures were adjusted for brightness and contrast in Adobe Photoshop.

## Results

### Mice With Skeletal Muscle-Restricted Expression of hSOD1 Develop Motor Deficits and Paralysis and Have a Shortened Lifespan

**Figure 1:**
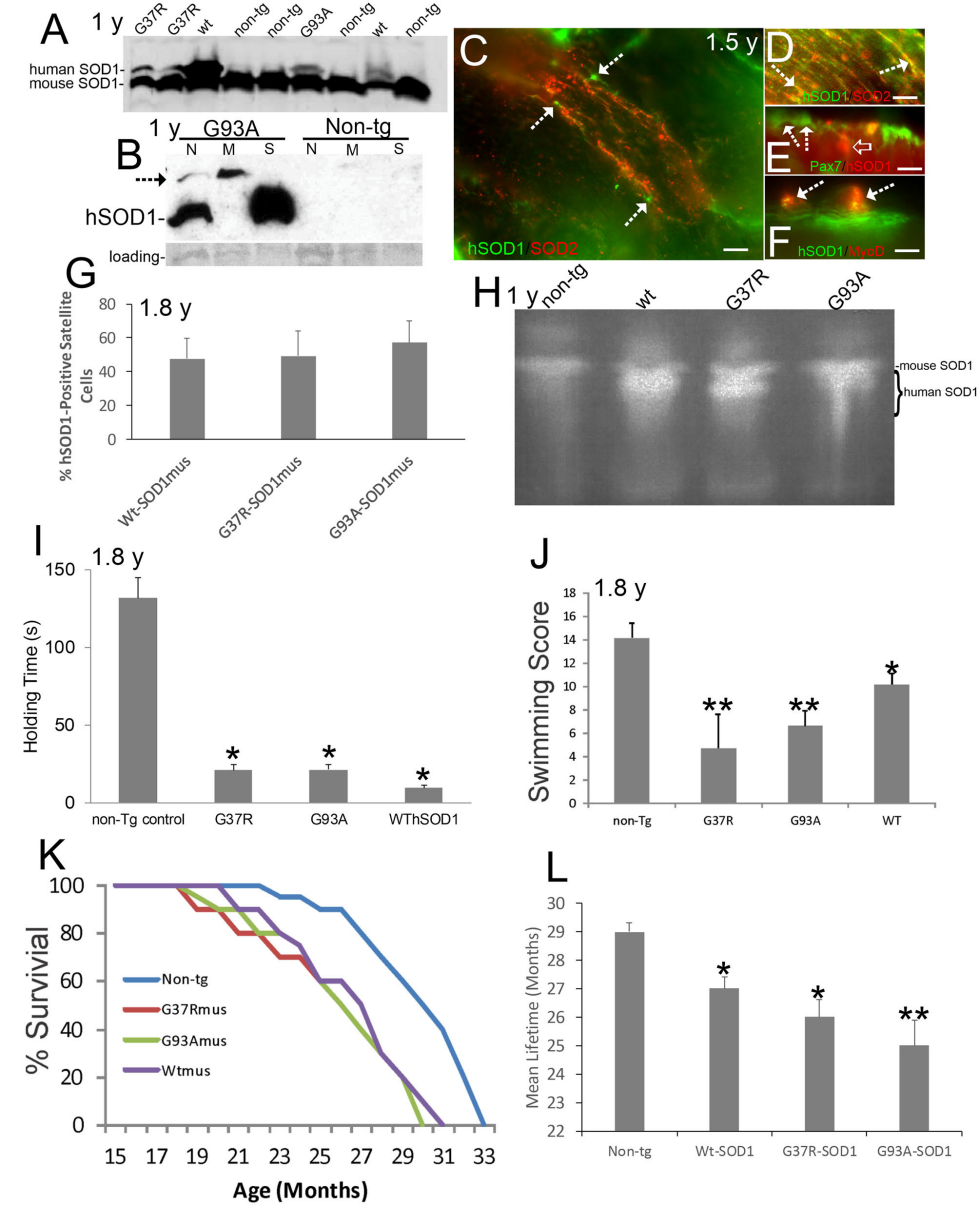
Verification and neurological phenotypes of hSOD1^mus^ mice. Ages are identified on panels where appropriate. **(A)** Western blot of mouse diaphragm extracts demonstrating the presence of Wt-, G37R-, and G93A-hSOD1 in tg mice but not in non-tg mice. An antibody that detects both mouse and hSOD1 was used. **(B)** Western blot of leg muscle subcellular fractions with hSOD1-specific antibody showing highest enrichment of monomer hSOD1 in the soluble fraction (S), lesser enrichment in the nuclear fraction (N), and barely detectable to low presence in the mitochondrial fraction. Oligomeric hSOD1 (arrow) was highest in the mitochondrial fraction, lesser in the nuclear fraction, and not seen in the soluble fraction. Extracts for non-tg skeletal muscle were negative for hSOD1. Ponceau staining of membrane shows protein loading. **(C)** Localization of hSOD1 (green) and mitochondrial marker SOD2 (red) in hSOD1^mus^ mouse myofibers. hSOD1 was localized diffusely throughout myofibers and was found at the myofiber periphery apparently localizing the satellite cells (arrows). Scale bar = 22.5 μm. **(D)** Some hSOD1 was localized to mitochondria (arrows, yellow). **(E,F)** hSOD1 was present in satellite cells (**E**, arrows) and co-localized with skeletal muscle satellite cell markers (**E**, Pax7, yellow cell) and MyoD (**F**, arrows). hSOD1 also formed cytoplasmic intramyofiber inclusions (**E**, open arrow, red). Scale bars = 9 μm. **(G)** Graph showing the percentages of satellite cell positive for hSOD1 in tg mice. Values are mean ± SD. **(H)** Enzymatic activity gel for SOD showing the functional activity of hSOD1 in tg mouse skeletal muscle. **(I)** Muscle strength holding time test showed marked weakness in hSOD1^mus^ mice (^*^p < 0.001). **(J)** Swimming testing showed significant deficit (^*^p < 0.05) in Wt-hSOD1^mus^ mice and greater deficit (^**^p < 0.01) in G37R- and G93A-hSOD1^mus^ mice. **(K)** Kaplan-Meier curves showing the survival of non-tg mice and hSOD1^mus^ mice. **(L)** Graph of mean (± SD) lifespan of non-tg and hSOD1^mus^ mice (^*^p < 0.05; ^**^p < 0.01).

We created and characterized at young ages tg mice expressing wild-type (WT)-, G37R-, and G93A-hSOD1 gene variants only in skeletal muscle (50, 66). These mice express hSOD1 transgene variants at ~ 50–110% of endogenous mouse SOD1 in striated skeletal muscles (50, 66), importantly the diaphragm (Figure 1A). With previous earlier generations of tg mice, we showed that hSOD1 was not in any spinal cord or brain neural cells and other organ systems and tissue types (50, 66). In subsequent later generations, the restricted expression of hSOD1 in skeletal muscle and the absence of hSOD1 in brain and spinal cord is maintained (Supplementary Figure 1). One tissue type was a notable exception. hSOD1 was detected in the heart of one WT-hSOD1 line and one G37R-hSOD1 line at old age (Supplementary Figure 1).

Subcellular fractions of skeletal muscle revealed hSOD1 in the soluble protein compartment (Figure 1B), as anticipated (8), and in nuclear and mitochondrial-enriched compartments (Figure 1B). In the mitochondrial-enriched fraction of ~15-month-old mice, hSOD1 was mostly present in an oligomer form (Figure 1B, arrow). hSOD1 was found as monomer and oligomer in the nuclear compartment (Figure 1B) as was seen in 8–12-month-old mice (72). Subcellular fractions of non-tg mouse skeletal muscle had no detectable bands of any size, demonstrating the transgenic state of the hSOD1^mus^ mice and specificity of the antibody for detecting hSOD1 as monomers and oligomers.

Immunofluorescence for hSOD1 in tg mouse skeletal muscle revealed hSOD1 in the myofiber cytoplasm as diffuse and particulate immunoreactivities (Figure 1C). Within myofiber cytoplasm, hSOD1 had partial colocalization with mitochondrial marker SOD2 (Figure 1D), consistent with the presence of hSOD1 in the mitochondrial fraction determined by western blotting (Figure 1B). Some hSOD1 immunoreactivity was visualized as large apparent inclusions (Figure 1E). hSOD1 also was present in apparent satellite cells (Figure 1C). This was confirmed by hSOD1 colocalization with satellite cell markers (65) Pax7 (Figure 1E) and MyoD (Figure 1F). hSOD1 was present in ~ 50% of skeletal muscle satellite cells in all tg mouse lines (Figure 1G). SOD activity gels demonstrated protein functional enzyme activity in skeletal muscle above that of mouse SOD1 in all hSOD1 tg variants (Figure 1H).

Our previous work on the anatomical and histological pathology of our hSOD1^mus^ tg mice focused on subjects at 1.0–1.5 years of age (50, 66). Here, we study these mice at mostly 1.5 years of age to end of life. As expected for longitudinal studies of mouse aging, various cancers were seen in older hSOD1^mus^ tg and non-tg mouse cohorts, but mice with confounding co-morbidities were excluded from this analysis. No histopathological studies are reported here on the identities of these cancers. Mice that were studied here were cancer-free, defined as no overt enlargement, malformation, discoloration, liquefaction, and hardening of any organ, including thymus and lymphoid tissues, spleen, lung, liver, gut, mesenteries, kidney, connective tissues, ovary, and testes.

hSOD1^mus^ mice at 1.5–1.8 years showed significant (*p* < 0.001) deficits in motor function detected in the holding/hanging test (Figure 1I) and significant (*p* < 0.01) deficits in a swimming test (Figure 1J). Earlier generations of younger mice also showed deficits on the holding/hanging tests and on running tests (50). Aging affected mice showed progressive limb weakness and paresis with motor deficits, paralysis, and shortened lifespan (Figure 1K; Supplementary Video 1). All hSOD1^mus^ tg mice became clinical with weakness and limb paresis; however, the paralysis phenotype was not fully penetrant, consistent with some human ALS patient cohorts (73). About 37% of mice became fully paralyzed between 1.5 years to end of life (Figure 1K and Supplementary Video 1). In all tg lines, lifespan was significantly (*p* < 0.05 or 0.01) shortened 10–16% (Figure 1L).

### The Skeletal Muscle System in hSOD1^mus^ tg Mice Develops Prominent Disease in Late Life

**Figure 2:**
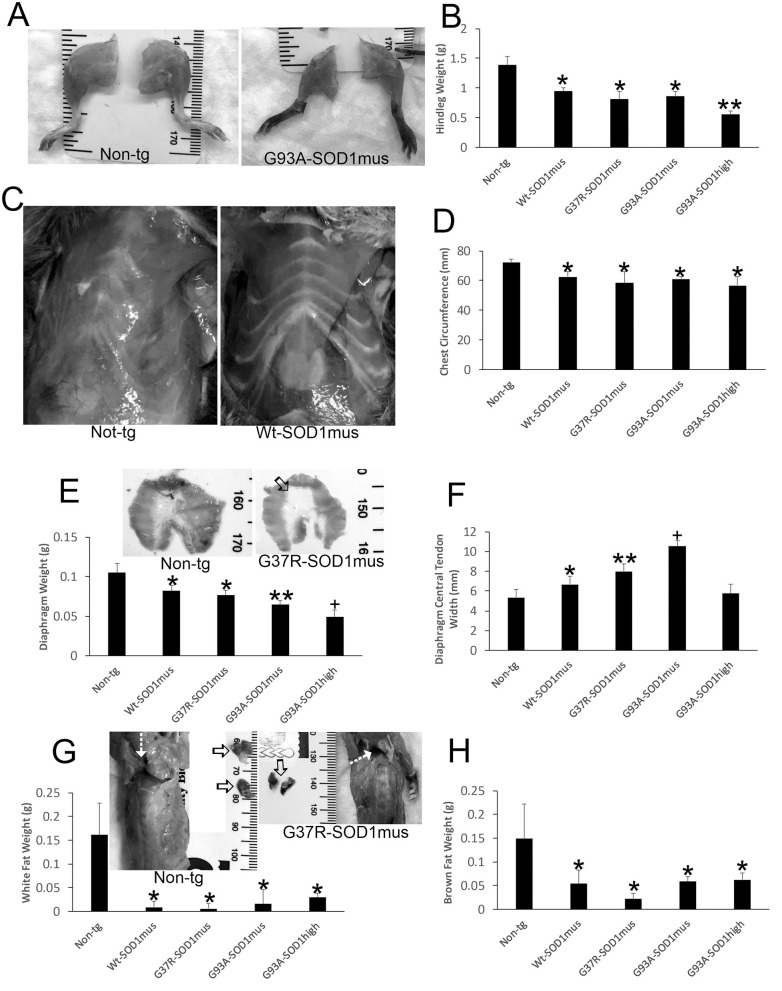
Gross anatomical pathology of hSOD1^mus^ mice. **(A)** Hindlegs of non-tg mice and hSOD1^mus^ mice showing the gross atrophy of the latter. **(B)** Graph of hindleg weights (mean ± SD) in age-matched non-tg mice and hSOD1^mus^ mice. Non-age-matched G93A-hSOD1^high^ expressing mice (74) were used as a comparator (^*^p < 0.01; ^**^p < 0.001). **(C)** Ventral view of the thorax of age-matched non-tg and hSOD1^mus^ mice showing pectoral and intercostal muscle wasting. **(D)** Graph of chest circumference (mean ± SD) in age-matched non-tg mice and hSOD1^mus^ mice ^*^p < 0.05. **(E)** Isolated diaphragms of non-tg mice and hSOD1^mus^ mice showing the gross atrophy and graph of diaphragm weights. Non-age-matched hSOD1-G93A^high^ expressing mice were used as a comparator (^*^p < 0.05; ^**^p < 0.01; ^+^*p* < 0.005). **(F)** Graph of diaphragm central tendon widths (mean ± SD) in age-matched non-tg mice and hSOD1^mus^ mice (^*^p < 0.05; ^**^p < 0.01; ^+^*p* < 0.005). **(G,H)** Images showing the backs of non-tg and hSOD1^mus^ mice. Hatched arrows identify interscapular regions. Isolated interscapular white adipose tissue (WAT) and brown adipose tissue (BAT) of non-tg mice (left, open arrows) and hSOD1^mus^ mice (right, open arrows) showing the gross atrophy and graphs of weights. WAT **(G)** was significantly depleted in all lines (^*^p < 0.001). BAT was significantly depleted in all lines (^*^p < 0001).

hSOD1^mus^ tg mice at 1 year of age showed subtle skeletal muscle atrophy, myofiber loss, and histochemical changes in ATPase and cytochrome c oxidase (50). At 2.0–2.25 years of age the body skeletal musculature appeared wasted in hSOD1^mus^ tg mice (Figures 2A,C,E). Hindlegs were grossly atrophied and reduced in mass compared to old age-matched non-tg littermate mice (Figure 2B). G93A-hSOD1^mus^ mice were the most severely affected hSOD1^mus^ tg mice (Figure 2B) but were not as affected as the non-conditional G93A-hSOD1^high^ tg mouse line (Figure 2B). All major leg muscle groups were affected (Figure 2B). The ribcages of all hSOD1^mus^ tg mouse lines were grossly atrophic and showed reductions in low thorax circumference measured at the xiphoid process (Figures 2C,D). The isolated diaphragms of hSOD1^mus^ tg mice had reduced mass at mid-stage to end-stage disease (Figure 2E), and striated muscle flanks of the diaphragm were replaced by expansion of non-myofiber central tendon (Figure 2F).

### The Adipose System in hSOD1^mus^ tg Mice Is Abnormal

In our previous studies (50, 66), we did not work on the body adipose system. However, full necropsies of our mice were done during later disease. During the gross pathological examinations of hSOD1^mus^ tg mice and age-match non-tg mice, differences in body fat distributions were observed. Both white adipose tissue (WAT) and brown adipose tissue (BAT) appeared attritional in tg mice compared to non-tg mice. WAT appeared depleted in many areas throughout the body of old hSOD1^mus^ tg mice at end-stage disease, including the legs, thorax, and back (Figure 2G). The back was particularly noteworthy for the loss of interscapular WAT mass (Figure 2G). While examining interscapular WAT deposits, the deeper BAT was found overtly attritional with loss of mass in hSOD1^mus^ tg mice (Figure 2H).

### Skeletal Myofiber Degeneration, Mitochondriopathy, and Satellite Cell Apoptosis Are Significant in hSOD1^mus^ tg Mice

**Figure 3:**
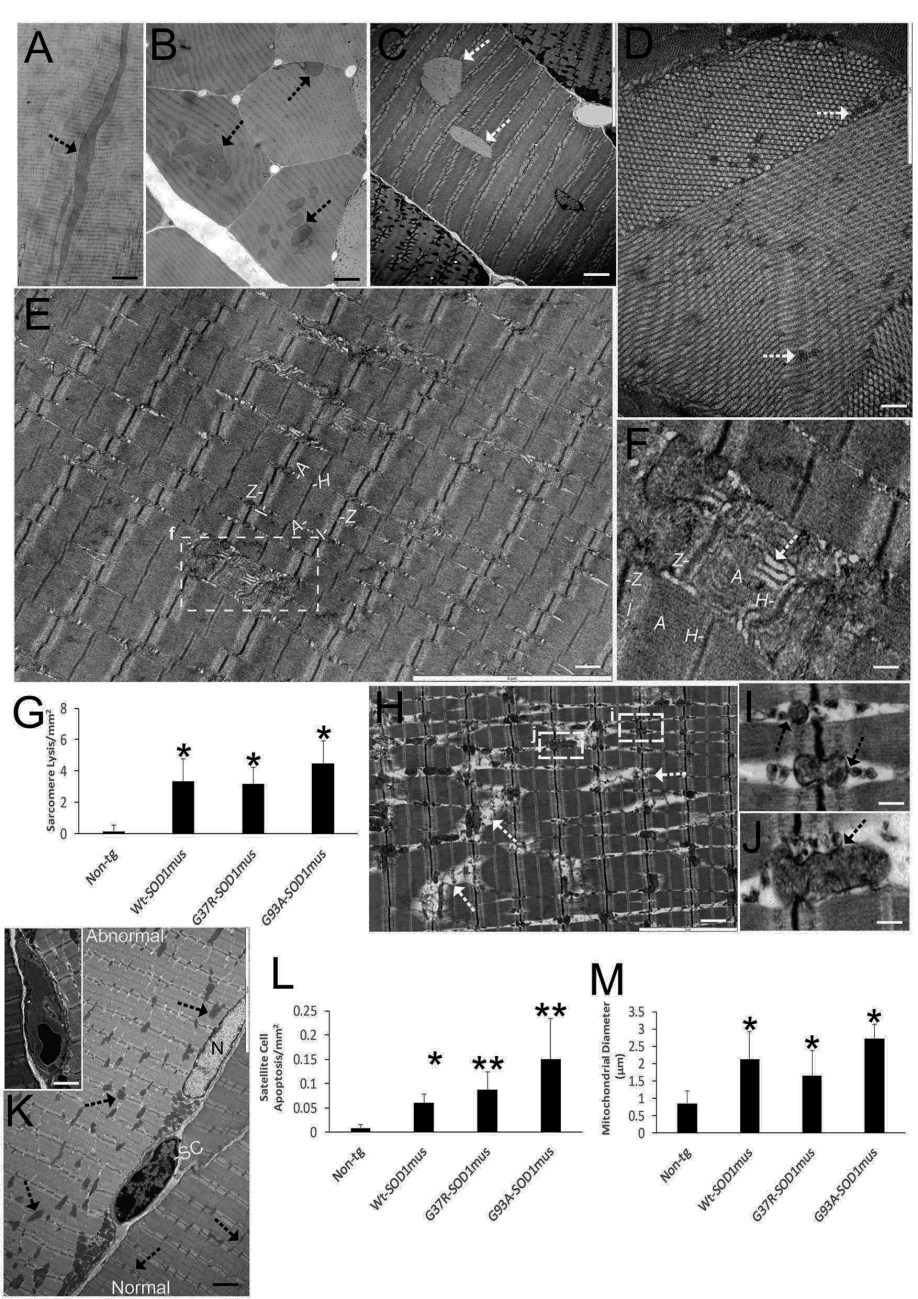
EM shows that skeletal muscle of hSOD1^mus^ mice develops myofiber inclusions, sarcomere degeneration, mitochondriopathy, and satellite cell apoptosis. **(A,B)** Plastic sections (0.5 μm thick) showing intramyofibrillar inclusions (arrows) in longitudinal **(A)** and cross-section **(B)** profiles. Scale bars = 225 μm **(A)** and 75 μm **(B)**. **(C)** Electron micrograph of hSOD1^mus^ mouse with two intramyofiber inclusions (arrows). Scale bar = 4.5 μm. **(D)** Electron micrograph of myofiber showing the crystalline-like arrays containing islands of cytoplasm (arrows). Scale bar = 2.25 μm. **(E)** Low magnification micrograph of a myofiber showing the sarcomere arrangement with distinguishing Z-bands, I-bands, A-bands, and H zones. Box of injured sarcomere is shown in **(F)**. Scale bar = 3 μm. **(F)** Early individualized sarcomere destruction is typified by dissolution of clear band patterns and sarcoplasmic reticulum cistern swelling (white arrow). Scale bar = 0.5 μm. **(G)** Graph of swollen/lytic sarcomeres (mean ± SD) in gastrocnemius of age-matched non-tg mice and hSOD1^mus^ mice (^*^p < 0.01). **(H)** Electron micrograph of many sarcomeres with advanced lysis (arrows) and mitochondriopathy (boxes shown in I and J). Scale bar = 2.25 μm. **(I,J)** Z-band mitochondria vesiculation (arrows). Scale bar = 0.4 μm. **(K)** Satellite cell (SC) nascent apoptosis as suggested by the chromatin clumping and nuclear envelop margination. A normal myonucleus is nearby (N). The myofiber on left has florid mitochondrial swelling (arrows), while the myofiber on right has normal appearing mitochondria (arrows). Inset shows a fully apoptotic SC. Scale bar = 11 μm (inset, 4.5 μm). **(L)** Satellite cell apoptosis (mean ± SD) in gastrocnemius of age-matched non-tg mice and hSOD1^mus^ mice (^*^p < 0.05; ^**^p < 0.01). **(M)** swollen mitochondria (seen as mitochondrial diameters) (mean ± SD) in gastrocnemius of age-matched non-tg mice and hSOD1^mus^ mice (^*^p < 0.01).

We used plastic sections (0.5 μm thick) and EM to directly identify skeletal muscle degeneration in hSOD1^mus^ tg mice (Figure 3). Inclusion formation, sarcomere singularities, mitochondriopathy, and satellite cell apoptosis were prominent phenotypes of these tg mice seen at higher resolution. Intramyofibrillar inclusions seen in longitudinal profile were up to 100 μm in length (Figure 3A). In cross-sectional profile, intramyofibrillar inclusions were 5–20 μm in width and seen as clusters (Figure 3B). EM revealed that these inclusions were crystalline lattices with elementary units arrayed as groupings in different planes (Figures 3C,D). Another prominent aspect of disease in hSOD1^mus^ tg mice was damage to individual sarcomeres extending from Z-band to Z-band (Figures 3E–H). Sarcomeres appeared swollen with banding disruption and tubule dilation and vesiculation in H zones (Figure 3F) and Z-bands (Figure 3H). This pathology was found in all hSOD1^mus^ tg mouse genotypes (Figure 3G) but was negligible in non-tg age-matched control mice (Figure 3G). In previous work, we found that subsarcolemmal mitochondrial numbers were lower in all three genotypes of hSOD1^mus^ tg mice at 15 months of age (66). Here, we show that disease also manifests in skeletal muscle as mitochondriopathy seen as mitochondrial vesiculation (Figures 3I,J) and swelling (Figures 3K,M). Lastly, satellite cell apoptosis was significant in all hSOD1^mus^ tg mouse genotypes (Figures 3K,L).

**Figure 4:**
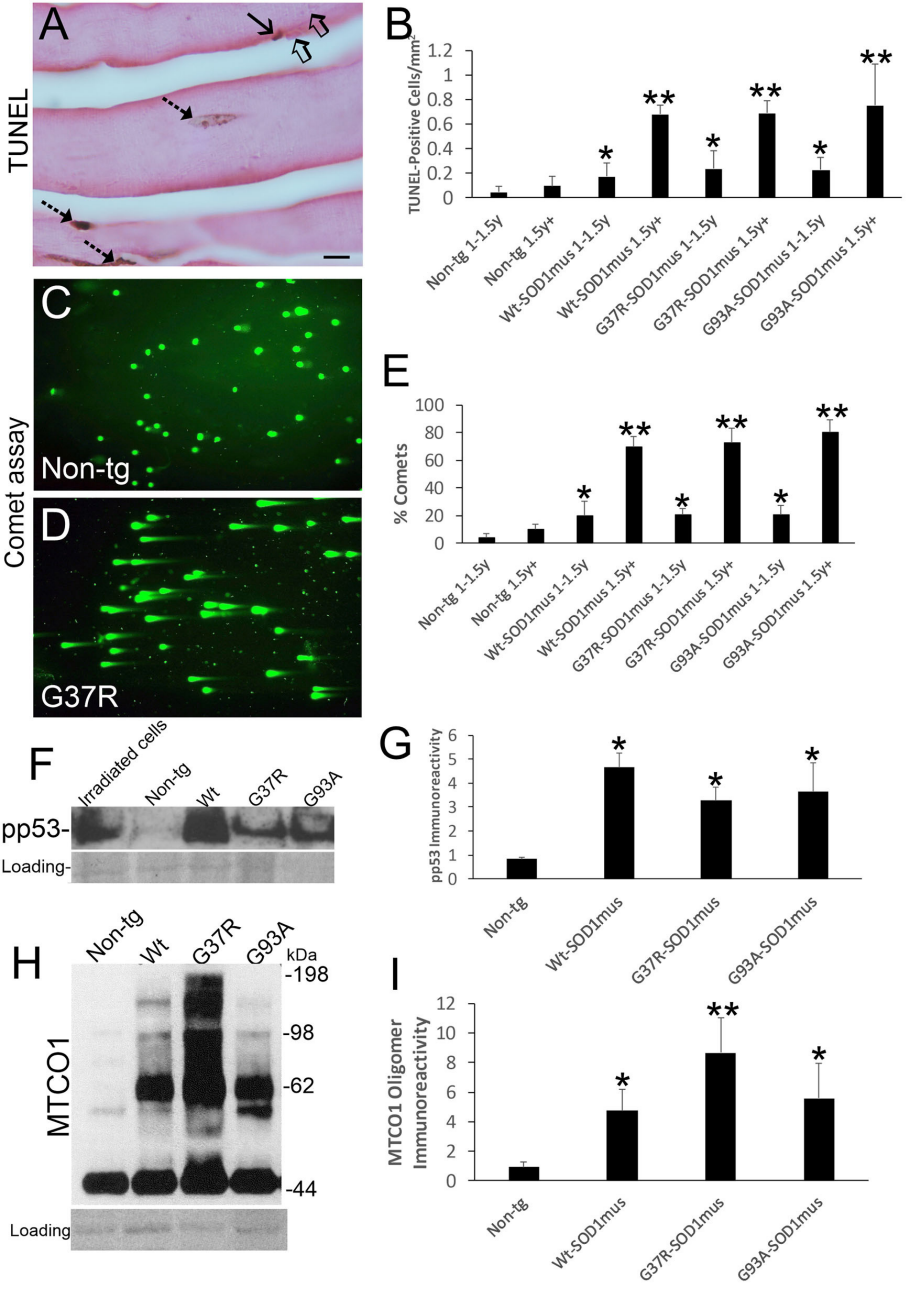
DNA damage, p53 activation, and mitochondrial complex IV proteinopathy occur in skeletal muscle of hSOD1^mus^ mice. **(A)** TUNEL visualized DNA-DSB accumulation in myonuclei (hatched arrows) and a putative satellite cell (solid arrow). Unlabeled nuclei are present (open arrows). Scale bar = 15 μm. **(B)** TUNEL positive nucleus accumulation in skeletal muscle is age-related in hSOD1^mus^ mice (mean ± SD, ^*^p < 0.05; ^**^p < 0.001). **(C,D)** Alkaline comet assay shows marked age-related accumulation of DNA-SSBs (SYBR green comets) in hSOD1^mus^ mice compared to non-tg age-matched controls. **(E)** Graph showing the age-related accumulation of nuclei with DNA-SSBs (mean ± SD, ^*^p < 0.05; ^**^p < 0.01). **(F,G)** Western blotting shows prominent p53 activation in skeletal muscle of hSOD1^mus^ mice. **(H,I)** Western blotting shows prominent accumulation of aggregated mitochondrial cytochrome c oxidase subunit 1 (MTCO1). Ponceau S stained membrane shows protein loading. Graph shows quantification of MTCO1 high molecular species (^*^p < 0.01, ^**^p < 0.001).

We examined the histological and biochemical pathology of skeletal muscles in hSOD1^mus^ tg mice and age-matched non-tg mice. Previously, we qualitatively reported on myofiber cell death detected by TUNEL in 12-month-old mice (50). Here, we show in greater depth the TUNEL identified cell death of individual myofibers and satellite cells of hSOD1^mus^ tg mice (Figure 4A). TUNEL thus corroborated the EM findings (Figures 3K,L). The accumulation of TUNEL-positive nuclei in diaphragm was age-dependent (Figure 4B). While TUNEL detects DNA double-strand breaks (75), we used the alkaline comet assay to detect DNA-SSBs. DNA damage accumulation determined by comet assay was not part our previous work (50, 66). Florid DNA-SSBs accumulated in skeletal muscle as seen by comet profiles in hSOD1^mus^ tg mice but not in age-matched controls (Figures 4C,D). The accumulation of nuclei in diaphragm with DNA-SSBs was age-dependent (Figure 4E). DNA-SSBs are potent inducers of p53 activation (76). Western blotting of skeletal muscle extracts identified significant increases in the levels of phosphorylated p53 (Figures 4F,G). These changes in DNA damage were antedated by high molecular weight aggregation of the complex IV enzyme cytochrome c oxidase subunit I (Figures 4H,I) in hSOD1^mus^ tg mice at non-symptomatic and early symptomatic disease compared to age-matched non-tg control littermate mice. Catalase enzyme activity in skeletal muscle was similar in age-matched non-tg mice and hSOD1^mus^ tg mice (data not shown).

### NMJ Pathology Emerges Early During Disease in hSOD1^mus^ tg Mice

**Figure 5:**
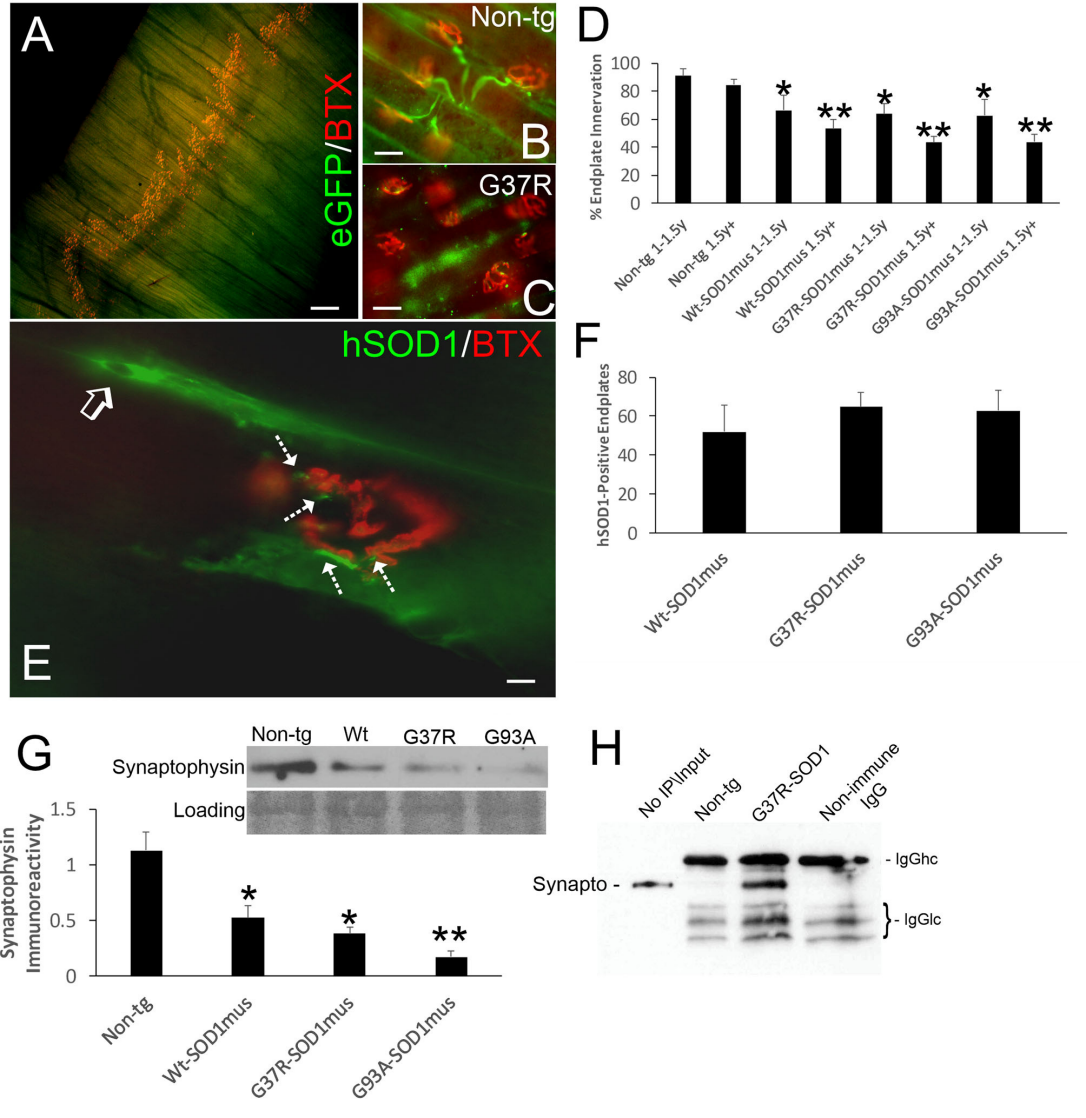
Structural and molecular pathology occur at the NMJ in skeletal muscle of hSOD1^mus^ mice. **(A)** Whole mount of a mouse diaphragm showing the distribution pattern of NMJ/motor endplates (red, α-bungarotoxin, BTX). Scale bar = 18 μm. **(B)** Motor endplate (red, BTX) innervation by ventral horn motor neuron axons (green, Hb9-egfp) in non-tg mouse diaphragm. Scale bar = 30 μm. **(C)** Motor endplate denervation and axonopathy (swollen dystrophic axons, green) in hSOD1^mus^ mouse diaphragm. Scale bar = 30 μm. **(D)** Graph showing the age-related loss of diaphragm motor endplate innervation (mean ± SD, ^*^p < 0.05; ^**^p < 0.01). **(E)** hSOD1 aggregates (arrows, green) near motor endplates. **(F)** About 50% of motor endplates are associated with hSOD1 aggregates by midlife in hSOD1^mus^ mouse diaphragm. Scale bar = 9 μm. **(G)** Western blotting revealed a loss of presynaptic terminal synaptophysin in hSOD1^mus^ mouse diaphragm. Ponceau S stained membrane shows protein loading. **(H)** Immunoprecipitation of synaptophysin in diaphragm extract followed by western blotting for 3-nitrotyrosine shows increased nitration of synaptophysin in hSOD1^mus^ mouse but comparatively sparse in age-matched non-tg mice.

We evaluated NMJ integrity using histological and biochemical approaches. The diaphragm of hSOD1^mus^ tg mice at different stages of disease and age-matched non-tg mice was examined using motor endplate visualization with BTX and motor neuron axon innervation of the endplate by neurofilament immunofluorescence and genetic expression of eGFP (Figures 5A–C). Previously we evaluated endplate occupancy at 12 months of age and found a reduction in G37R-hSOD1^mus^ mice (50). Here we confirm this finding and further show this NMJ abnormality in older mice of all our hSOD1^mus^ lines. Significant unoccupancy of motor endplates was observed beginning around 1–1.5 years of age in G37R-, G93A-, and WT-hSOD1^mus^ tg mouse genotypes (Figure 5D), and denervation was progressive through older ages (Figure 5D). hSOD1 immunoreactivity was detected at ~50% of motor endplates (Figures 5E,F), and, unlike the diffuse cytoplasmic labeling seen within myofibers, hSOD1 at motor endplates appeared aggregated (Figure 5E). Western blotting of skeletal muscle extracts detected significant loss of presynaptic marker synaptophysin (Figure 5G). Immunoprecipitation of skeletal muscle synaptophysin followed by 3-nitrotyrosine western blotting identified nitrated synaptophysin accumulation in hSOD1^mus^ tg mice but not in age-matched non-tg mice (Figure 5H). Skeletal muscle calcineurin and rapsyn did not show increased nitration in hSOD1^mus^ tg mice (data not shown).

**Figure 6:**
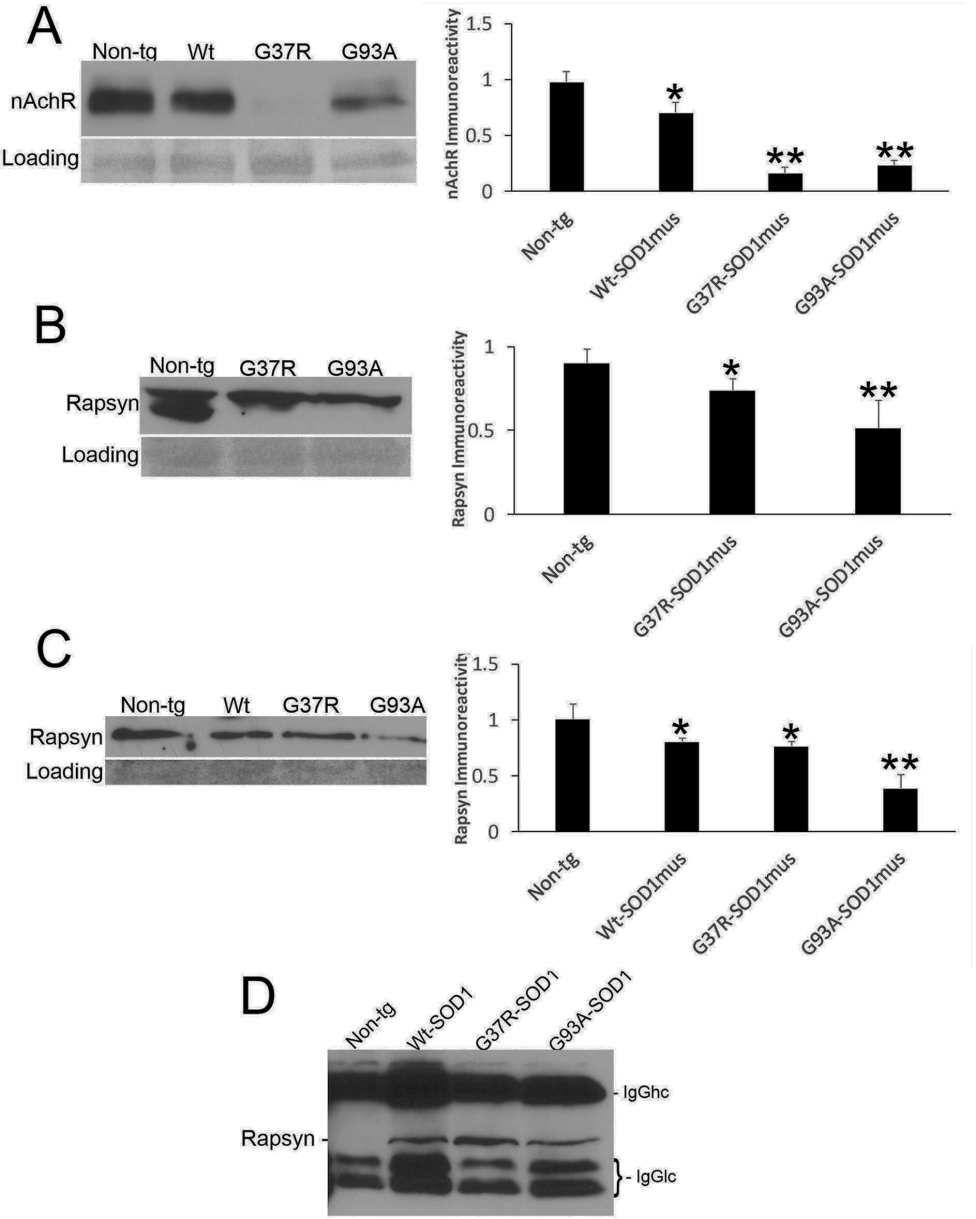
Molecular pathology is prominent at the postsynaptic compartment of the NMJ of hSOD1^mus^ mice. **(A)** Western blotting showed depletion of nicotinic acetylcholine receptor (nAchR) in hindleg skeletal muscle of WT, G37R, and G93A mutant mice (mean ± SD, *p* < 0.05; ^**^p < 0.01). Ponceau S stained membrane shows protein loading. **(B)** Scaffold protein rapsyn in diaphragm of G37R and G93A mutant mice (mean ± SD, *p* < 0.05; ^**^p < 0.01). Ponceau S stained membrane shows protein loading. **(C)** Western blotting showed depletion of the scaffold protein rapsyn in hindleg skeletal muscle of WT, G37R, and G93A mutant mice (mean ± SD, ^*^p < 0.05; ^**^p < 0.01). Ponceau S stained membrane shows protein loading. **(D)** Immunoprecipitation of hSOD1 in hindleg skeletal muscle extracts followed by western blotting for rapsyn showed co-precipitation of hSOD1 and rapsyn in tg mice.

Because NMJ presynaptic anomalies were observed in hSOD1^mus^ tg mice, and hSOD1 was detected at motor endplates (Figure 5E), we examined NMJ postsynaptic components (Figure 6). Loss of postsynaptic NMJ marker nicotinic acetylcholine receptor (nAchR) (Figure 6A) was detected within 12–15 months of age in hSOD1^mus^ tg mice compared to age-matched non-tg mice. Loss of NMJ scaffold protein rapsyn (Figure 6B) was seen in the diaphragm (Figure 6B) and hindleg muscles (Figure 6C) in hSOD1^mus^ tg mice at the same age. Co-immunoprecipitation identified that hSOD1 interacts with rapsyn (Figure 6D), consistent with the visualization of hSOD1 at motor endplates and its apparent aggregation (Figure 5E).

### NO Signaling Is Upregulated in Skeletal Muscle of hSOD1^mus^ tg Mice

**Figure 7:**
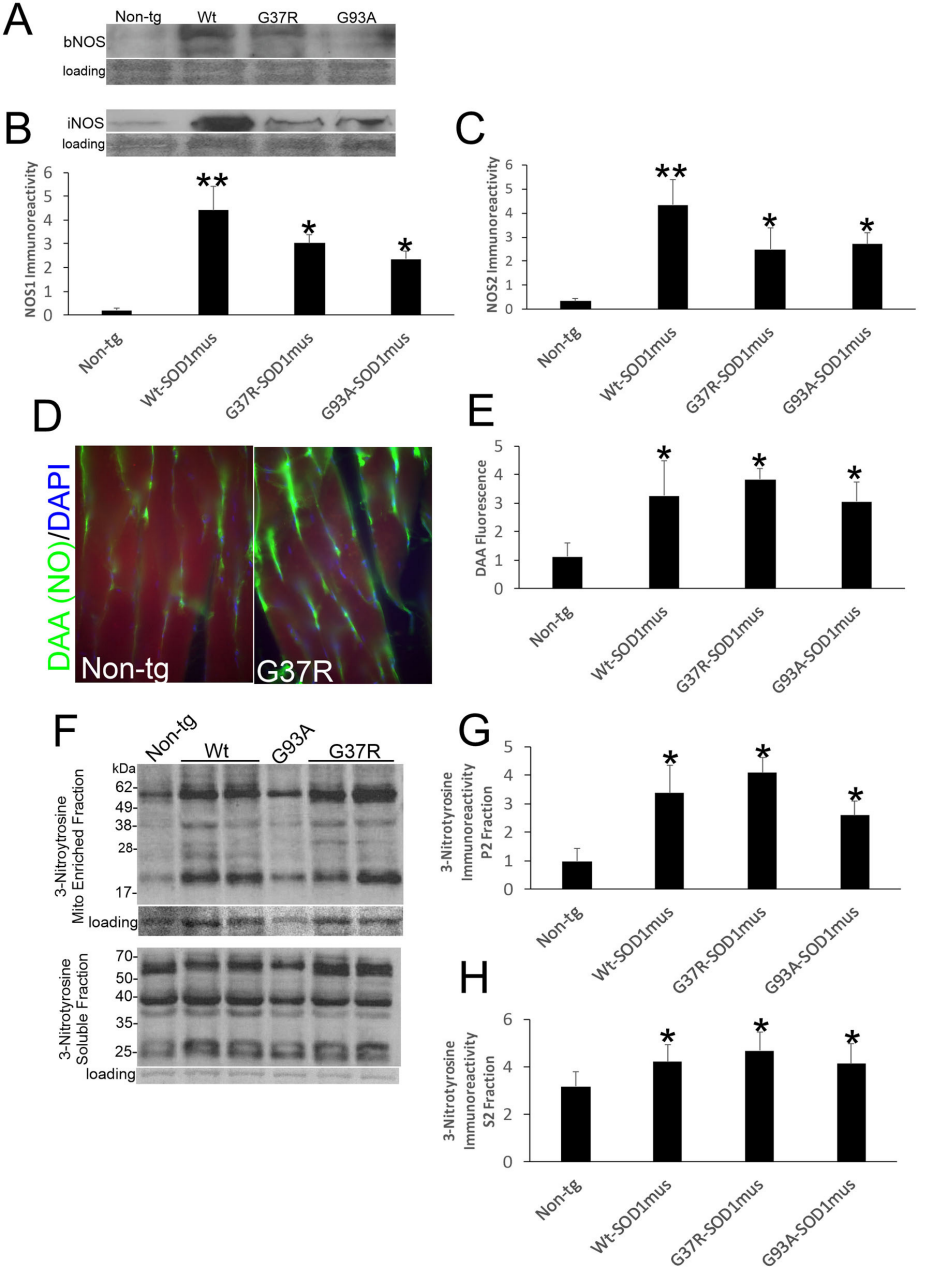
Nitric oxide signaling is upregulated in skeletal muscle of hSOD1^mus^ mice. **(A,B)** Western blotting showed upregulation of NOS1 (bNOS) and NOS2 (iNOS) in hindleg of hSOD1^mus^ mice. Ponceau S stained membrane shows protein loading. **(B)** Graph for NOS1 immunodensity demonstrating significantly elevated levels in Wt, G37R, and G93A tg mice (mean ± SD, ^*^p < 0.01; ^**^p < 0.001). **(C)** Graph for NOS2 immunodensity demonstrating significantly elevated levels in Wt, G37R, and G93A tg mice (mean ± SD, ^*^p < 0.01, ^**^p < 0.005). **(D)** *In vivo* imaging of NO production (green) with DAA injection into gastrocnemius of non-tg and G37R- hSOD1^mus^ mice. Scale bar = 45 μm. **(E)** Graph demonstrating significantly elevated DAA fluorescence in gastrocnemius sections of Wt, G37R, and G93A tg mice. **(F)** Western blotting showed increased protein 3-nitrotyrosine immunoreactivity in mitochondrial (Mito)-enriched fractions and in soluble fractions of tg mice. Ponceau S stained membrane shows protein loading. **(G,H)** Graphs demonstrating significantly elevated protein nitration in the mitochondrial-enriched protein fraction **(G)** and soluble protein fraction **(H)**. Graphs demonstrating significantly elevated protein nitration in hindleg of Wt, G37R, and G93A tg mice (mean ± SD, ^*^p < 0.05).

Western blotting detected significant increases in the levels of NOS1 and NOS2 in skeletal muscle extracts of hSOD1^mus^ tg mice at 12–15 months of age compared to age-matched non-tg mice (Figures 7A–C). All hSOD1 variants showed an upregulation (Figures 7B,C). *In situ* assessments of NO production by injections of DAA into skeletal muscle and thorax of live mice followed by a 24 h survival revealed increased NO production in the gastrocnemius and diaphragm of hSOD1 mice compared to age-matched non-tg mice (Figures 7D,E). To confirm this *in vivo* result, we fingerprinted peroxynitrite production by western blotting for protein nitration. Protein nitration in soluble protein and mitochondrion-enriched fractions was increased in WT-, G37R-, and G93A-hSOD1^mus^ tg mice between 12 and 15 months of age compared to age-matched non-tg mice (Figures 7F–H).

### hSOD1^mus^ tg Mice Develop ALS-like Pathology in Spinal Cord and MN Loss

**Figure 8:**
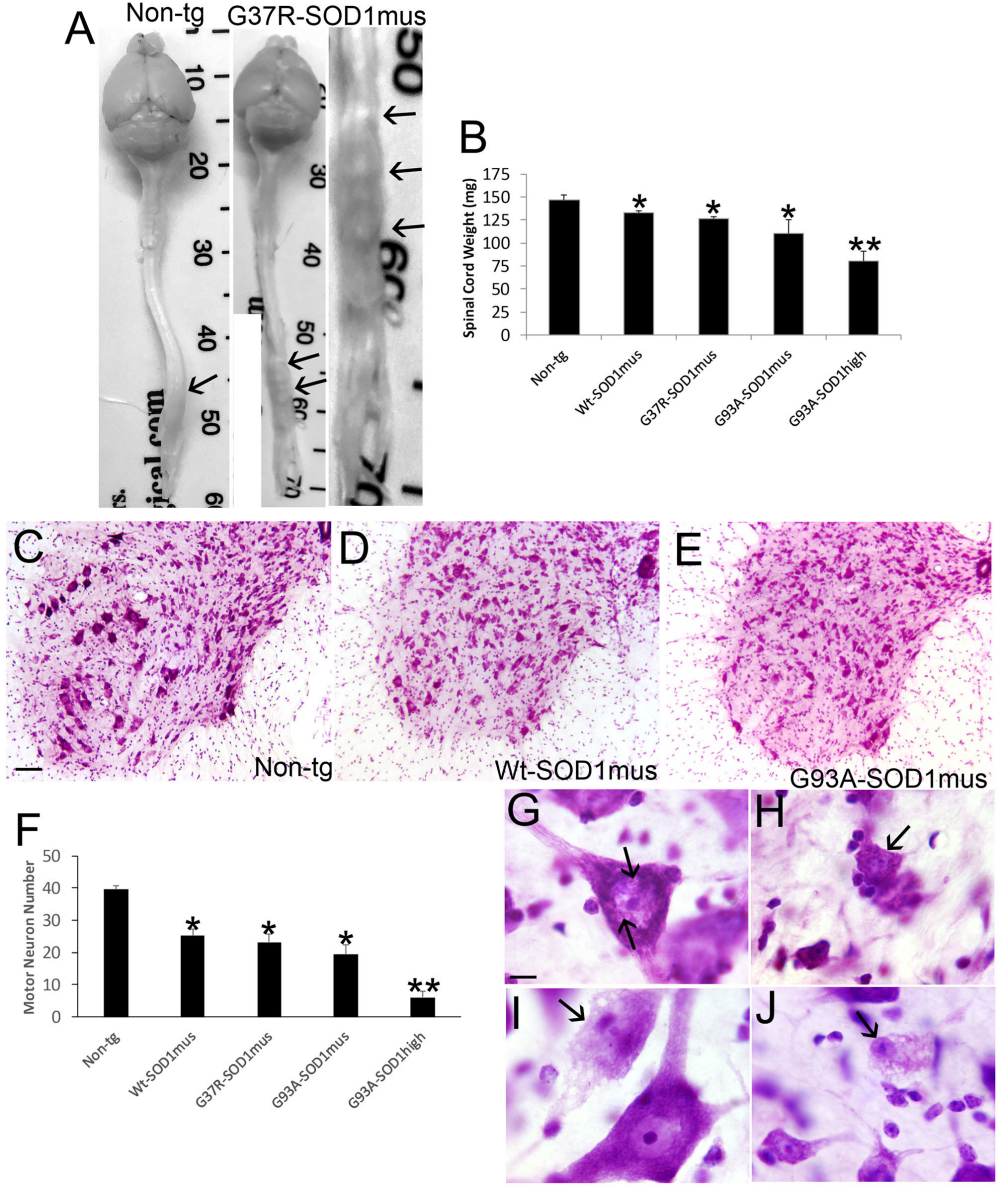
Spinal cord atrophy and motor neuron degeneration occur in hSOD1^mus^ mice. **(A)** Isolated brain and spinal cord of age-matched non-tg and G37R-hSOD1^mus^ mice. Lumbar spinal cord enlargements are identified (arrows). The non-tg cord is smooth and uniformly tapering. The tg cord is irregular, atrophic, and nodular (right panel). **(B)** Graph of spinal cord weights (mean ± SD) in age-matched non-tg mice and hSOD1^mus^ mice at endstage disease. Non-age-matched G93A-hSOD1^high^ expressing mice (74) were used as a comparator (^*^p < 0.01; ^**^p < 0.001). **(C–E)** Representative examples of Nissl-stained lumbar spinal cord sections of age-matched non-tg and hSOD1^mus^ (Wt and G93A variants) mice showing apparent loss of large motor neurons. Scale bar in C = 10.5 μm [same for **(D,E)**]. **(F)** Graph of lumbar spinal cord motor neuron counts at L4-5 (mean ± SD) in age-matched non-tg mice and hSOD1^mus^ mice at endstage disease. Non-age-matched G93A-hSOD1^high^ expressing mice (74) were used as a comparator (^*^p < 0.01; ^**^p < 0.001). **(G,H)** Nissl staining showing motor neurons in G37R- hSOD1^mus^ lumbar spinal cord with degenerative features, including small round nuclear inclusions [**(G)**, arrows], apoptotic somal attrition [**(H)**, arrow], cell body cytoplasm vacuolation with somal and nuclear attrition [**(I)**, black arrow], and cell body cytoplasm and dendrite vacuolation [**(J)**, arrow]. Scale bar in G = 7.5 μm [same for **(H–J)**].

Anatomic and microscopic neuropathology was done on the spinal cord of hSOD1^mus^ tg mice and age-matched controls (Figure 8). The spinal cords of hSOD1^mus^ tg mice at end-stage disease showed gross atrophy (Figure 8A), including prominent atrophy of nerve roots and nodulation in the lumbosacral region, and significant loss of total spinal cord (plus conus medullaris) mass (Figure 8B). Histologically, in cresyl violet-stained sections of lumbar spinal cord, counts of large, Nissl-rich, multipolar MNs were reduced 40–50% in each line (Figures 8C–F). Previously in younger mice at 12 months of age we found a lesser loss of MNs in G37R and G93A mice and no significant loss in WT-hSOD1^mus^ mice (50). Thus, the degeneration of MNs in WT-hSOD1^mus^ mice appears slower than in the mutant hSOD1 lines. Subsets of remaining spinal MNs contained nuclear inclusions (Figure 8G) and exhibited different forms of cell body degeneration including apoptotic-like attrition (Figure 8H) and necrotic-like vacuolation of the cell body and dendrites with apparently maintained somal size (Figure 8I) or somal atrophy (Figure 8J).

**Figure 9:**
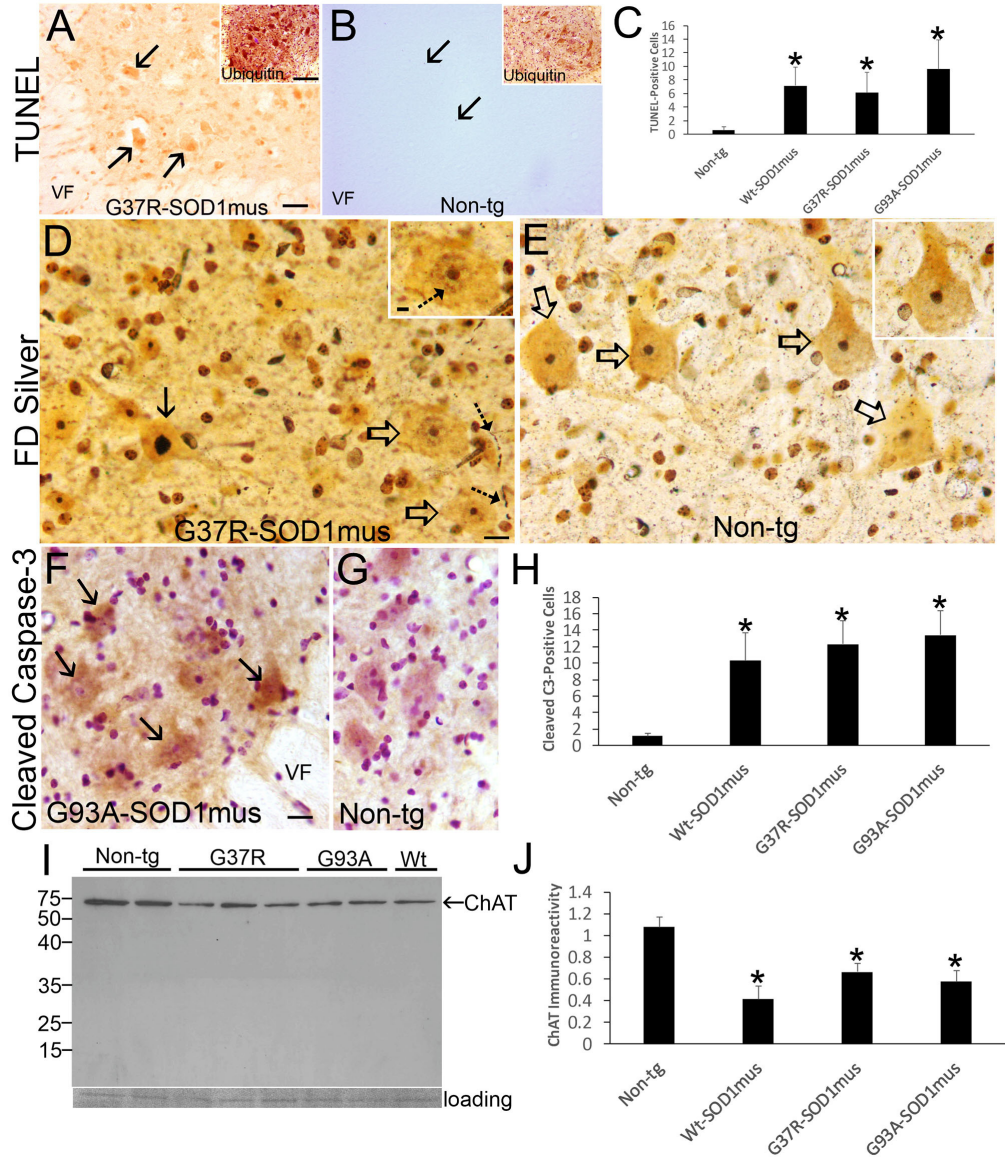
Motor neuron cell death in hSOD1^mus^ mice is an apoptotic-like process. **(A)** TUNEL visualized DNA-DSB accumulation in nuclei of dying motor neurons (arrows) in G37R-hSOD1^mus^ mouse lumbar spinal cord. Inset shows strong accumulation of ubiquitin in motor neurons. VF, ventral funiculus. Scale bar = 45 μm (same for B); A inset scale bar = 42 μm [same for **(B)** inset]. **(B)** Motor neurons in age-matched control mouse lumbar spinal cord were TUNEL-negative. A few TUNEL-positive small cell nuclei were detected (arrows). Inset shows ubiquitin immunoreactivity is less apparent compared to G37R-hSOD1^mus^ mouse [**(A)**, inset]. **(C)** Graph of lumbar spinal cord TUNEL-positive cells (all sizes) at L4-5 (mean ± SD) in age-matched non-tg mice and hSOD1^mus^ mice at endstage disease (^*^p < 0.01). **(D)** FD silver staining identified motor neurons in hSOD1^mus^ mice with apoptotic condensation of the nucleus (black arrow) as distinct from motor neurons with an open nucleus (open arrows), but these latter neurons also had nascent condensation of nuclear chromatin (inset, hatched arrow). Degenerating neurites were also present in the neuropil (hatched arrows). Scale bar in D = 15 μm [same for **(E)**]; D inset scale bar = 5 μm [same for **(E)** inset]. **(E)** In age-matched non-tg mice, motor neuron nuclei were essentially silver negative, except for the nucleolus (open arrows, and inset). **(F,G)** Immunohistochemistry showed cleaved caspase-3 immunoreactivity enrichment in hSOD1^mus^ mouse motor neurons **(F)**, with accumulation in the nucleus [**(F)**, arrows]. In age-matched control motor neurons, while sometimes showing cytoplasmic cleaved caspase-3, the nucleus was generally unlabeled **(G)**. VF, ventral funiculus. Scale bar in F = 22.5 μm [same for **(G)**]. **(H)** Graph of the number of lumbar motor neurons positive for nuclear cleaved caspase-3 in L4-5 (mean ± SD) of age-matched non-tg mice and hSOD1^mus^ mice at endstage disease (^*^p < 0.001). **(I,J)** Western blotting for the motor neuron marker ChAT **(I)** revealed prominent reductions in the levels in hSOD1^mus^ compared to controls [**(J)**, mean ± SD, ^*^p < 0.01].

MN loss in WT-, G37R-, and G93A-hSOD1^mus^ tg mice was confirmed with cell death determinations using *in situ* DNA fragmentation analysis (TUNEL), silver staining, immunostaining for cleaved caspase-3, and western blotting for the cholinergic MN marker ChAT (Figure 9). In spinal cord sections, large ventral horn cells were found to be TUNEL-positive in each mouse line (Figures 9A–C). MNs in hSOD1^mus^ tg mice were also strongly positive for ubiquitin (Figure 9A, inset) compared to age-matched control MNs (Figure 9A, inset). Silver staining identified MNs in tg mice with degenerating apoptotic-like nuclei (Figure 9D) that were not seen in age-matched control spinal cord (Figure 9E). Neuritic abnormalities were also seen in the spinal cord neuropil of tg mice (Figure 9D). MNs with less advanced degeneration showed silver-positive nuclear strands (Figure 9D, inset) that were not seen in the nucleus of control MNs (Figure 9E, inset). MNs positive for cleaved caspase-3 immunoreactivity were present in tg mouse spinal cord (Figures 9F,H) but infrequently in age-matched non-tg mice (Figures 9G,H). Consistent with MN loss seen histologically, ChAT level was reduced significantly in western blots of hSOD1^mus^ tg mouse spinal cord as early as mid-stage disease (Figures 9I,J). Previously we qualitatively described an apparent loss of ChAT in spinal cord by western blotting (50).

### hSOD1^mus^ tg Mouse Spinal MNs Develop TDP-43 Pathology, Axonopathy, and Mitochondriopathy

**Figure 10:**
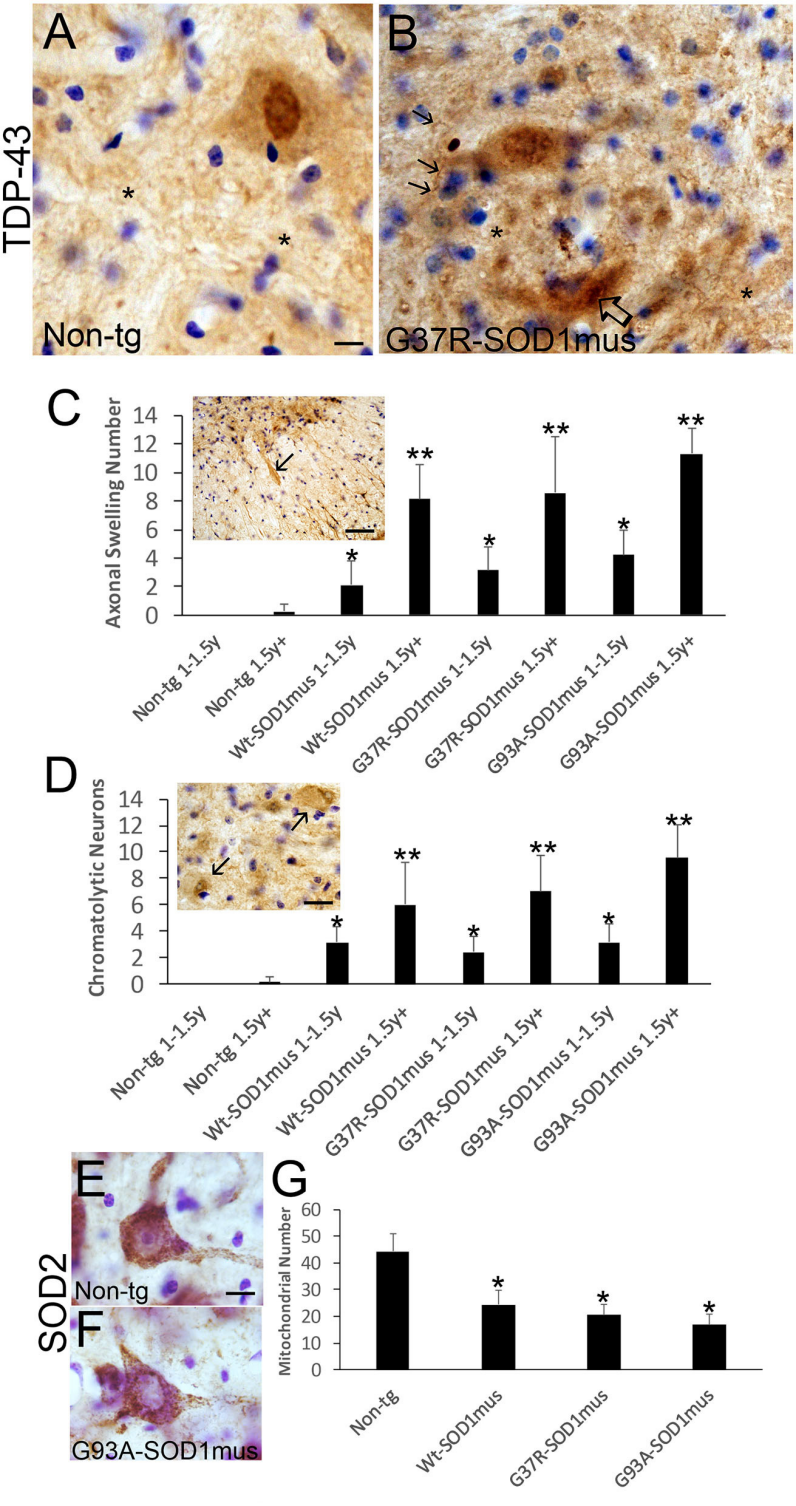
Motor neurons in hSOD1^mus^ mice develop TDP-43 abnormalities, axonopathy, and mitochondrial depletion. **(A,B)** TDP-43 immunostaining of motor neurons in non-tg **(A)** and hSOD1^mus^ **(B)** mice. At about midlife (12 months), in normal motor neurons **(A)**, TDP-43 is highly enriched in the nucleus compared to the cytoplasm, but in hSOD1^mus^ mice **(B)**, the distinction between distributions of TDP-43 immunoreactivity in the nucleus and cytoplasm becomes less obvious, and MNs in mutant mice form TDP-43 inclusions [**(B)**, open arrow]. In mutants, there is also enhanced immunoreactivity for TDP-43 in the neuropil (^*^) with the presence of neuritic abnormalities [**(B)**, arrows]. Scale bar in A = 7.5 μm [same for **(B)**]. **(C)** TDP-43-positive motor neuron axonal swellings develop in aging hSOD1^mus^ mice. Scale bar = 90 μm. Graph showing the age-dependent increase in axonal swellings (arrows) in hSOD1^mus^ mice (mean ± SD, *p* < 0.05; ^**^p < 0.01). **(D)** As axonal swellings accumulate, there is an age-related increase in the number of chromatolytic motor neurons seen by TDP-43 staining (arrows). Scale bar = 22.5 μm. **(E,F)** Mitochondrial SOD2 staining in hSOD1^mus^ mice is characterized by loss of mitochondrial density in the perikaryal-proximal dendrite domains. Scale bar = 15 μm. **(G)** Graph of mitochondrial number (mean ± SD) age-matched non-tg mice and hSOD1^mus^ motor neuron at endstage disease (^*^p < 0.01).

TDP-43 pathology was seen in hSOD1^mus^ tg mice but not in age-matched controls (Figure 10). In aged control mouse MNs, TDP-43 immunoreactivity was mostly in the nucleus, while cytoplasmic immunoreactivity was distinctly lower in comparison to the nucleus (Figure 10A), but in tg mouse MNs TDP-43 immunoreactivity was nuclear and, more prominently, cytoplasmic and was seen in the spinal cord neuropil (Figure 10B). hSOD1^mus^ tg mice formed large swollen and dystrophic axons that were prominent in the ventral funiculus near the ventral root exit zones (Figure 10C, inset) and were positive for TDP-43 (Figure 10C, inset). Consistent with axonopathy was the accumulation of chromatolytic MNs seen by Nissl staining and TDP-43 (Figure 10D). In chromatolytic MNs, TDP-43 immunoreactivity was mostly cleared from the cytoplasm (Figure 10D). Perikaryal mitochondria, detected by SOD2 immunostaining, were depleted in hSOD1^mus^ MNs (Figures 10E–G).

### hSOD1^mus^ tg Mouse Spinal MN Cell Death Signatures Are Consistent With DNA Damage-Driven Apoptosis

**Figure 11:**
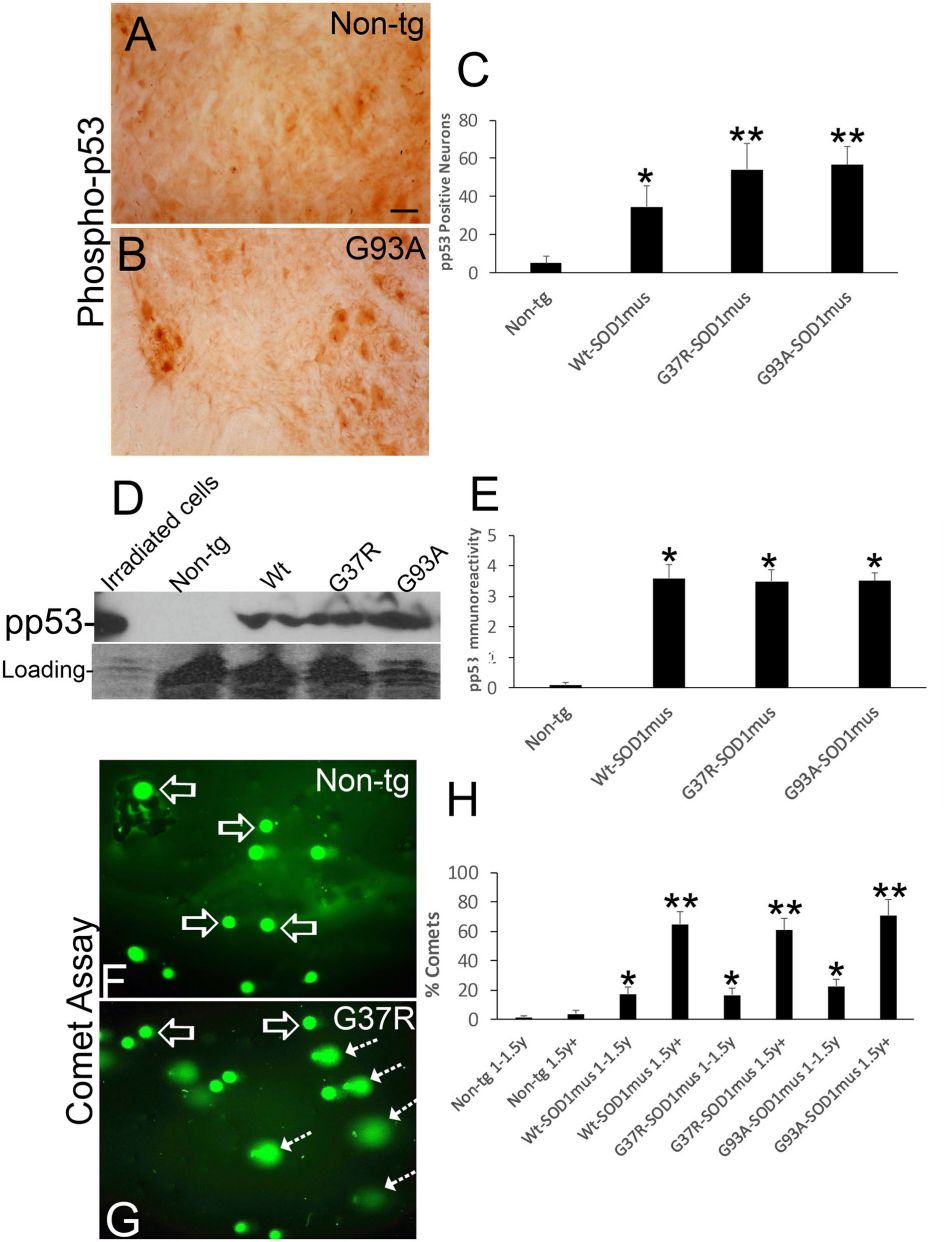
DNA damage and p53 activation occur in motor neurons of hSOD1^mus^ mice. **(A,B)** Immunohistochemistry showing apparent accumulation of p-p53 positive spinal motor neurons in hSOD1^mus^ mice [**(B)**, arrows] compared to age-matched controls **(A)**. Scale bar in A = 22.5 μm [same for **(B)**]. **(C)** Graph of p-p53 positive spinal motor neurons (mean ± SD) in age-matched non-tg mice and hSOD1^mus^ motor neurons at 1.5–2 years of age (^*^p < 0.01, ^**^p < 0.001). **(D)** Western blotting showed upregulation of p-p53 in spinal cord of hSOD1^mus^ mice. **(E)** Graph of p-p53 immmunoreactivity (mean ± SD) in western blots of age-matched non-tg and hSOD1^mus^ spinal cord at midlife (^*^p < 0.001). **(F,G)** Alkaline comet assay on isolated spinal motor neurons shows marked presence of DNA-SSBs (SYBR green comets) in hSOD1^mus^ mice compared to non-tg age-matched controls. **(H)** Graph showing the age-dependent accumulation of motor neurons with nuclear DNA-SSBs (mean ± SD, *p* < 0.05; ^**^p < 0.01).

Group VII, VIII, and IX MNs were found in various stages of chromatolysis (Figure 10D) that is typical of axonal injury (Figure 10C) and generally antecedent to retrograde apoptosis of MNs in rodent models (77, 78) and, putatively, in human (79). We found that subsets of ventral horn MNs were immunopositive for phosphorylated p53 (Figures 11A–C), and western blotting identified significant increases in phospho-p53 in spinal cord homogenates of hSOD1^mus^ tg mice compared to age-matched non-tg littermates (Figures 11D,E). Alkaline comet assays on isolated lumbar MNs from mid-stage (1.5 years) and end-stage (2^+^ years) disease hSOD1^mus^ mice showed significant age-dependent accumulation of DNA-SSBs compared to spinal MNs of non-tg age-matched control mice (Figures 11F–H).

## Discussion

Previously we have shown that skeletal muscle-restricted expression of hSOD1 can cause MN degeneration (50), but this earlier study was not a longitudinal aging study of clinical and pathological outcomes. Here, we extend our studies of mechanisms of ALS with the major finding that restricted expression of WT and mutant (G37R, G93A) hSOD1 in skeletal musculature is enough to cause an age-related ALS-like phenotype in older mice. The disease phenotype included a plethora of clinical, anatomic pathology and histopathological, and biochemical features. Clinically, mice developed deficits in motor function, some developed fulminant paralysis, and lifespan was shortened compared to non-tg mice. The anatomic pathology was wasting of the somatic musculature, including the diaphragm, attrition of adipose tissue, and spinal cord atrophy. Histopathologically, these mice had myofiber and satellite cell degeneration, myofiber inclusions, mitochondriopathy, and loss of diaphragm motor innervation; furthermore, in spinal cord, MNs were depleted, formed cytoplasmic and nuclear inclusions and TDP-43 pathology, accumulated cleaved caspase-3, and exhibited florid axonopathy. Biochemically, skeletal muscles had an upregulation of NOS isoforms and enhanced NO production; molecular dismantling of the NMJ, including presynaptic and postsynaptic pathology; skeletal muscle nuclei accumulated DNA-SSBs; and activated p53. Similarly, in spinal cord, MNs accumulated DNA-SSBs and activated p53. This work shows that hSOD1 in skeletal muscle is a driver of disease in mouse ALS and identifies a non-autonomous mechanism for MN degeneration explaining their selective vulnerability as likely being a form of target deprivation-induced retrograde neurodegeneration.

These hSOD1^mus^ tg mice thus develop a robust disease phenotype of neurologic, anatomic, and histopathologic abnormalities reminiscent of human ALS (1). Importantly, they develop older-age fatal disease that includes paralysis, wasting of the somatic skeletal musculature, and MN degeneration. hSOD1^mus^ tg mice could be a physiologically more relevant small animal model of ALS than other hSOD1 tg mouse models reported to date when considering their low hSOD1 transgene copy number (50), clinical course, and pathology. Other tg hSOD1 mice with lifespans longer than the most commonly used G93A^high^ copy number mouse (74) are G37R mice (72, 80), but these mice cannot be used to identify CNS vs. skeletal muscle disease causality because both tissues express the mutant hSOD1 transgene. The prolonged disease course in our mice could be related to the level of restricted expression of hSOD1 in different skeletal muscle groups which might be insufficient to drive rapid onset and clinical course of disease. However, we suspect that the level of hSOD1 expression in diaphragm is a critical determinant of lifespan because we have seen some hSOD1^mus^ tg mice become moribund with possible respiratory problems and die at 1.0–1.5 years of age without overt wasting of skeletal musculature and adipose tissue or other forms of overt disease. Leaky promoter effects in heart in some tg hSOD1^mus^ lines is also a consideration. A possible respiratory phenotype in our mice is interesting because some human ALS cases have respiratory onset disease (4). Another possibility is that the endogenous satellite cell-driven regenerative capacity of skeletal muscle is enough to prolong the disease course in our mice, despite the presence of hSOD1 in satellite cells, and thus making aging a critical risk factor for disease onset because reserve and resiliency is depleted. These effects in our mice would be relevant physiologically to human ALS with an onset age range of 47–52 years for fALS and 58–63 years for sporadic ALS (1, 4).

Thus, disease in skeletal muscle could be a pathologic trigger in ALS as suggested by others (81). In tg non-conditional G93A-hSOD1 mice, muscle hypertrophy induced by muscle-specific expression of IGF-1 conveys significant extension of life (49), and muscle IGF-1 variant attenuates the pathology in spinal cord (82). We find that WT and mutant (G37R and G93A) hSOD1 is toxic to skeletal muscle cells *in vivo*. The precise toxic mechanisms are still uncertain. Our EM work showed the formation of large intramyofibrillar crystalline-like inclusions very similar to those found in inclusion body disease (83). We also identified in these mice individualized sarcomere-scale destruction with sentinel events occurring in the sarcoplasmic reticulum t-tubule/triad system and mitochondria. Diseased intramyofibrillar mitochondria showed significant swelling and extraordinary vesiculation and apparent extrusion of cristae. We reported previously subsarcolemmal mitochondrial accumulation in younger mice (66). A nuclear presence of hSOD1 in myonuclei and satellite cells also could be part of the disease instigating process. Nuclear localization of hSOD1 in spinal MNs has been seen in tg mouse models with CNS expression (72). Nuclear hSOD1 can impact transcriptional control (84) and DNA repair (85). Our data suggest the possibility that sequestration of rapsyn from the NMJ by hSOD1 interaction might be a sentinel, progressive and age-related, event in the disease linking skeletal to spinal cord by causing a postsynaptic molecular dysfunction of nAchR leading to motor endplate loss of function. A hSOD1-rapsyn interaction is present in 12–15-month-old mice, but we are unsure its inception. Interestingly while NMJ proteins were depleted, NOS1 and NOS2 were upregulated. This upregulation could be meaningful to ALS pathogenesis because genetic deletion of NOS2 (64) and systemic pharmacological inhibition of NOS2 (86) extend the lifespan of non-conditional hSOD1-G93A tg mice. Importantly, this NOS upregulation could drive the increased NO production in skeletal muscle that we have discovered. These events may promote aberrant oxidative chemistry, evidenced by increased protein carbonyl formation (50), and nitrative stress evidenced here by increased protein nitration in different subcellular fractions, synaptophysin nitration in diaphragm, and DNA damage accumulation. However, these events seem to be occurring in the absence of changes in catalase. NO is a potent inducer of DNA-SSBs and DNA-DSBs in MNs (70). Because there is no hSOD1 in spinal MNs or in other CNS cells in this mouse system, yet spinal MNs degenerate and subsets (40–50%) are eliminated, the disease instigation is likely extrinsic to MNs and could involve NO in skeletal muscle.

There is growing evidence that WT SOD1 can be pathogenic in ALS (7, 12, 13, 87–89). Interestingly, our WT hSOD1^mus^ tg mice developed disease. There is precedent for WT hSOD1 causing disease. In human ALS tissues, misfolded SOD1 has been detected in sporadic ALS (87). In cell culture, oxidized WT hSOD1 can cause dose-dependent cell death, like mutant hSOD1 variants (12). Moreover, zinc deficiency can render WT SOD1 toxic to MNs in culture through mechanisms involving a gain in redox reactivity and catalysis of tyrosine nitration in proteins (13). Our tg mice expressing WT and mutant hSOD1 show evidence for oxidative stress and severe protein nitration in skeletal muscle consistent with the possibility that WT hSOD1 might acquire toxic properties through redox chemistry, thereby contributing to skeletal muscle damage, and thus MN disease. However, WT hSOD1 presence in mouse skeletal muscle did not significantly alter the catalase component of the antioxidant network.

Retrograde neurodegeneration of MNs could be an integral part of ALS pathogenesis in a target deprivation type of scenario (75, 90). Neurogenic denervation atrophy could also play into this pathological process (91) once disease is instigated in MNs through retrograde signaling mechanisms involving mitochondria (64) and perturbations in neurotrophin signaling (49, 92). Primary pathology in skeletal muscle, including NMJ-motor unit injury, could set up the target deprivation of MNs (93). Distal NMJ dismantling has garnered considerable attention as a mechanism of disease in ALS (93–95). The MN pathology seen here, and shown elsewhere (50, 57, 70), including axonopathy, chromatolysis, perinuclear mitochondrial abnormalities, DNA-SSB accumulation, p53 activation, and cell death, are consistent with distal axon-target pathology and target deprivation. Thus, MN degeneration in hSOD1^mus^ tg mice might follow muscle disease and be a form of retrograde dying-back, apoptotic-like degeneration with similarities to human ALS (90, 96). These findings add to the justification of revisiting neurotrophins and exploring microneurotrophins for the treatment of ALS (97).

We studied interscapular WAT and BAT in hSOD1^mus^ tg mice and found a loss in older life. This result is interesting because higher pre-diagnostic body fat (98), subcutaneous fat (99), and serum leptin, an energy regulating hormone produced by adipocytes (100), are all associated with decreased risk of ALS clinically. Several epidemiological dietary studies of populations in different countries show that higher fat consumption decreases risk of developing ALS (101–104). In the commonly used disease-aggressive non-conditional G93A-hSOD1 tg mouse model, high-fat diets extend lifespan (38, 105). While more work needs to be done (106), evidence is accruing suggesting that human ALS is a systemic metabolic and energy imbalance disorder (34), including data showing that the expenditure of energy exceeds energy uptake, possibly causing depletion of fat stores (101), and that most ALS patients are hypolipidemic (107) and have perturbed carbohydrate metabolism (108). Skeletal muscle (psoas) is hypermetabolic in ALS (109). Studies in rodents after axotomy and target deprivation have shown that neurons, including MNs, become hypermetabolic at the chromatolytic stage of degeneration (70, 78, 110); moreover, spinal MNs in non-conditional G93A-hSOD1 tg mice show high metabolic activity prior to mitochondrial failure and MN degeneration (64). During disease and body stress when energy demands are high and fuels are low, the skeletal musculature is a critical regulator of energy metabolism in many organs because of its ability of mobilize amino acids through proteolysis and to regulate glucose homeostasis (111). Our finding connects, albeit through uncertain mechanisms, the presence of ALS-causing hSOD1 only in the skeletal musculature and interscapular WAT and BAT in tg mice. A change in BAT is significant because this adipose organ is closely related to skeletal muscle, functions in thermogenesis and glucose homeostasis, and is enriched in mitochondria (112), introducing the possibility that the skeletal musculature and BAT may both be targets for disease modification and therapeutics in ALS. Interestingly, more physically fit men have a higher risk of dying at an early age from ALS than men with less physical capacity (113).

p53 has been implicated in the mechanism of MN degeneration in ALS (79, 114) (Martin and Liu, 2002). We found in hSOD1^mus^ tg mice, p53 activation in skeletal muscle and spinal cord. We also found the accumulation of DNA-SSB in myofiber nuclei and MN nuclei in these mice. Given that DNA-SSBs are a potent stimulus for p53 activation (76), these results are congruent with recent observations that DNA damage response and repair mechanisms are strongly activated in human ALS MNs in postmortem tissues and in human iPS cell-derived MNs with fALS SOD1 mutations (114).

## Conclusions

We tested the hypothesis that skeletal muscle is a primary site of pathogenesis in ALS that triggers MN degeneration by creating tg mice expressing WT, G37R-, and G93A-human *SOD1* gene variants only in skeletal muscle. The mice developed phenotypes, including shortened lifespan, anatomical pathology of skeletal muscle, adipose tissue, and spinal cord, and a microscopic pathology involving skeletal muscle stem cell apoptosis, aberrant mitochondria, and sarcomere degeneration, thus showing that hSOD1 in skeletal muscle is a driver of pathogenesis in ALS. The spinal MN degeneration involved inclusion formation, axonopathy, mitochondriopathy, DNA damage accumulation, and p53 activation and apoptosis suggesting a non-autonomous skeletal muscle driven mechanism for MN degeneration in ALS explaining their selective vulnerability as a form of target-deprivation retrograde neurodegeneration.

## Data Availability Statement

The original contributions presented in the study are included in the article/Supplementary Materials, further inquiries can be directed to the corresponding author/s.

## Ethics Statement

The animal study was reviewed and approved by JHU Animal Use and care Committee.

## Author Contributions

LM and MW: study concept and design, data acquisition and analysis, and manuscript writing. MW: tg mouse generation. All authors edited and approved the final version of the manuscript.

## Conflict of Interest

The authors declare that the research was conducted in the absence of any commercial or financial relationships that could be construed as a potential conflict of interest.

## Supplementary Material

Supplementary Figure 1 Western blot showing the presence or absence of hSOD1 and presence of mouse SOD1 in different tissues of hSOD1^mus^ tg mice in generation F10. A very long exposure is shown to ensure the absence of hSOD1 in some lanes. The positive control was a G93A-hSOD1^high^ expresser mouse line (G1H). hSOD1 was detected in skeletal muscle (hind leg) of all genotypes of hSOD1^mus^ tg mice. hSOD1 was not detected in CNS tissue (spinal cord). In two mouse lines (only one shown here) hSOD1 was detected in heart.

Supplementary Video 1 Video clip of a live mouse that is a wildtype (WT)- hSOD1^mus^ tg showing definite complete hind-limb paralysis and hindquarter and lower truncal muscle wasting and kyphosis.

## Abbreviations

ALS: amyotrophic lateral sclerosis
BAT: brown adipose tissue
DDR: DNA damage response
hSOD1: human superoxide dismutase
MN: motor neuron
NMJ: neuromuscular junction
NO: nitric oxide
tg: transgenic
WAT: white adipose tissue.
