# Supplementary figures and images for: Skeletal Muscle-Restricted Expression of Human SOD1 in Transgenic Mice Causes a Fatal ALS-Like Syndrome
Source: Front Neurol. 2020 Dec 14;11:592851. doi: 10.3389/fneur.2020.592851 (PMC7767933; doi:10.3389/fneur.2020.592851)

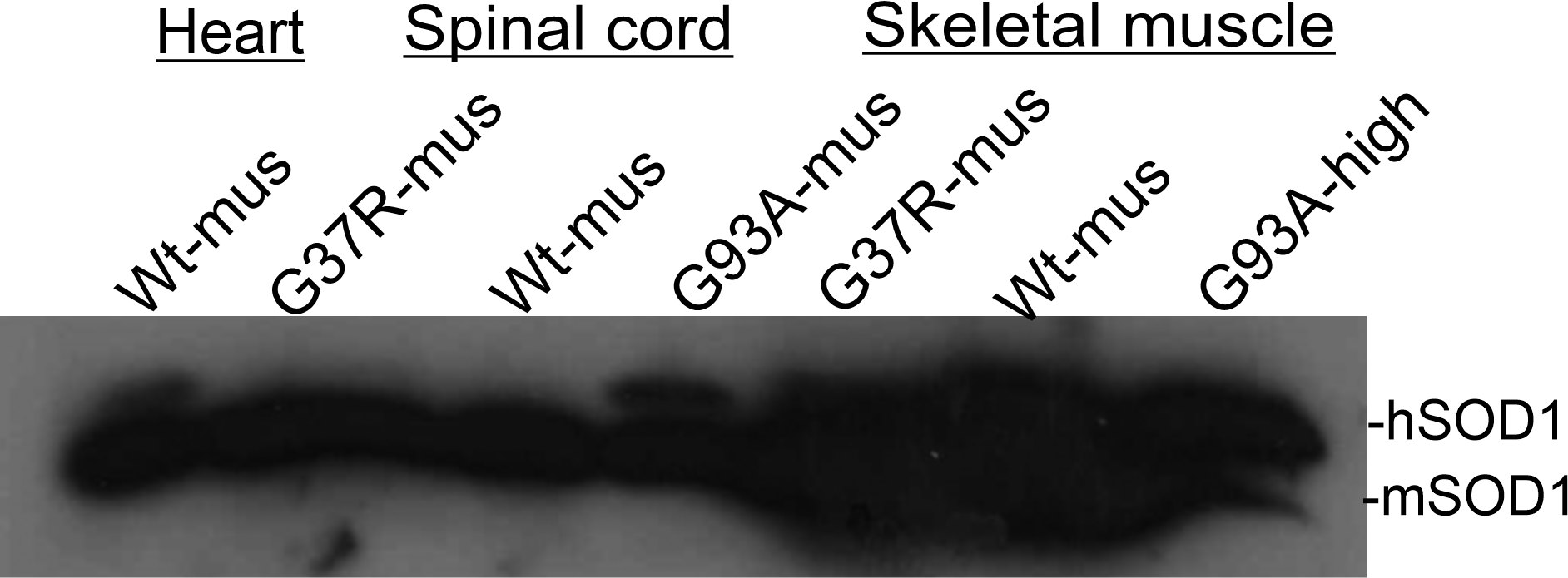

Supplement: Supplementary Figure 1 — Western blot showing the presence or absence of hSOD1 and presence of mouse SOD1 in different tissues of hSOD1mus tg mice in generation F10. A very long exposure is shown to ensure the absence of hSOD1 in some lanes. The positive control was a G93A-hSOD1high expresser mouse line (G1H). hSOD1 was detected in skeletal muscle (hind leg) of all genotypes of hSOD1mus tg mice. hSOD1 was not detected in CNS tissue (spinal cord). In two mouse lines (only one shown here) hSOD1 was detected in heart. [file Image_1.TIF]
